# Supplementary material for: Engineering bacterial thiosulfate and tetrathionate sensors for detecting gut inflammation
Source: Mol Syst Biol. 2017 Apr 3;13(4):923. doi: 10.15252/msb.20167416 (PMC5408782; doi:10.15252/msb.20167416)
Supplement: Supplementary file 1 — Appendix [file MSB-13-923-s001.pdf]

Appendix: Engineering bacterial thiosulfate and tetrathionate sensors for detection of gut inflammation

Kristina N.-M. Daeffler[1], Jeffrey D. Galley[2], Ravi U. Sheth[1], Laura C. Ortiz-Velez[2], Christopher O. Bibb[4], Noah F. Shroyer[3], Robert A. Britton[2], Jeffrey J. Tabor[1]

[1]Department of Bioengineering, Rice University, Houston, TX 77005, USA
[2]Department of Molecular Virology and Microbiology, Baylor College of Medicine, Houston, TX 77030, USA
[3]Department of Medicine, Baylor College of Medicine, Houston, TX 77030, USA
[4]Department of Pathology, Texas Children's Hospital, Houston, TX 77094, USA

Table of contents

Appendix Figure S1. *S. typhimurium* TtrS/TtrR domain layout and gene locus
Appendix Figure S2. Identification of P_{phsA} regulatory features
Appendix Figure S3. Plasmid maps for inducible ThsSR production
Appendix Figure S4. Shal_3129-induced activation of $P_{phsA342}$
Appendix Figure S5. Optimization of protein expression using inducible promoters to give the highest dynamic for ThsSR
Appendix Figure S6. Ligand inhibition assay for ThsSR
Appendix Figure S7. Schild plot analysis of ThsSR tetrathionate inhibition
Appendix Figure S8. Glucose sensitivity of thiosulfate-induced activation of ThsSR at P_{phsA}
Appendix Figure S9. Identification of $P_{ttrB344}$ regulatory features
Appendix Figure S10. Plasmid maps for inducible TtrSR production
Appendix Figure S11. Sbal195_3858-induced activation of $P_{TtrB344}$
Appendix Figure S12. Optimization of protein expression using inducible promoters to give the highest dynamic for TtrSR
Appendix Figure S13. Glucose sensitivity of tetrathionate-induced activation of $P_{ttrB185\text{-}269}$
Appendix Figure S14. TtrSR response in presence of 1 mM tetrathionate and 10 mM other TEAs
Appendix Figure S15. Optimization of the ThsSR for use in the mammalian gut
Appendix Figure S16. Optimization of TtrSR for use in the mammalian gut
Appendix Figure S17. Plasmid maps for constitutive ThsSR production used in *in* vivo studies
Appendix Figure S18. Plasmid maps for constitutive TtrSR production used in *in* vivo studies
Appendix Figure S19. Fluorophore maturation time course of anaerobically grown Nissle bacteria in PBS+1 mg/mL chloramphenicol
Appendix Figure S20. Flow cytometry profile before and after filtering through a 5 µM syringe filter
Appendix Figure S21. ThsSR performance in *ex vivo* ligated colon explants.
Appendix Figure S22. *S. baltica* TtrSR performance in *ex vivo* ligated colon explants.
Appendix Figure S23. ThsSR and TtrSR sensor performance with and without 3% DSS
Appendix Figure S24. Histograms of representative fecal samples from healthy control mice and DSS-treated mice
Appendix Figure S25. 2D flow cytometry data
Appendix Figure S26. Histologic scoring of all mice in the healthy (-DSS) and DSS-treated cohorts for wild type and inactivated mutant ThsSR and TtrSR strains
Appendix figure S27. Relationship between histologic score and fluorescence output of the ThsSR and ThsSR D57A sensors
Appendix Figure S28. Receiver operating characteristic curve for ThsSR performance.

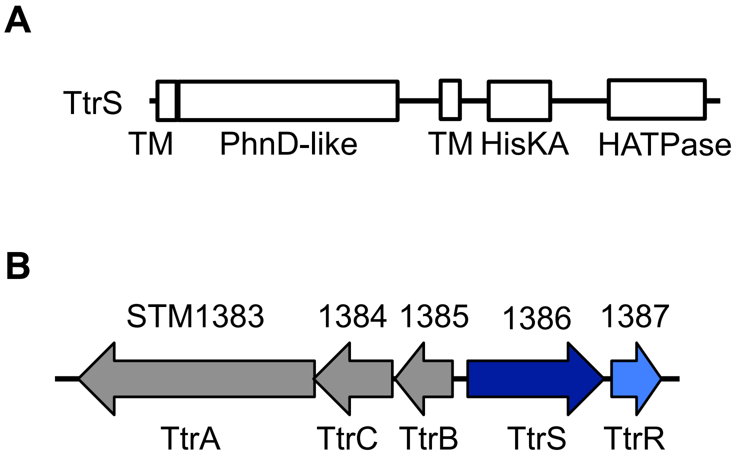

Appendix Figure S1. *S. typhimurium* TtrS/TtrR domain layout and gene locus. **(A)** Predicted domain architecture of TtrS. **(B)** Location of TtrSR (STM1386/7) and tetrathionate reductase, TtrBCA (STM1385-3), on the chromosome of *S. typhimurium* LT2.

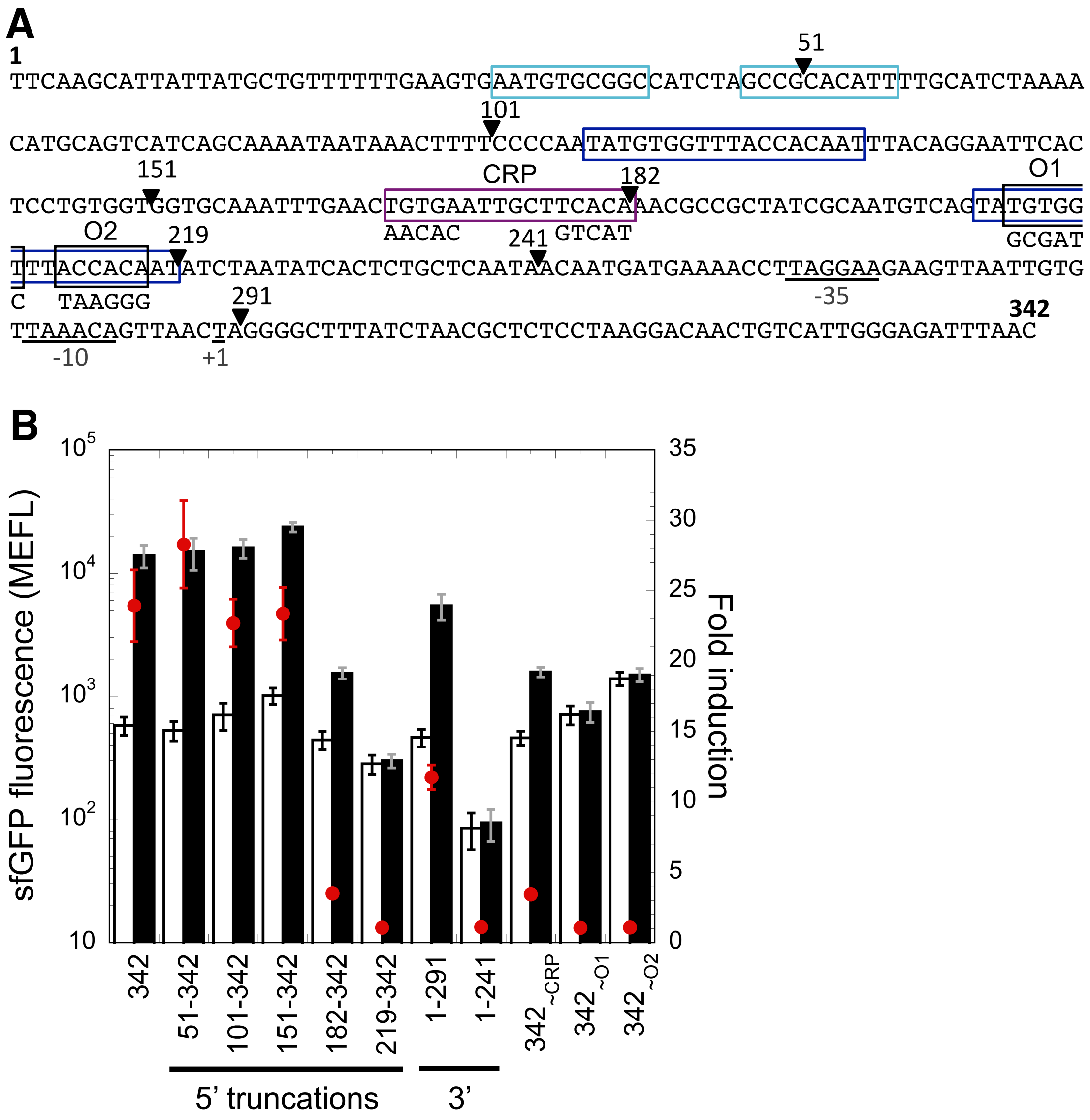

Appendix Figure S2. Identification of P_{phsA} regulatory features. **(A)** Two potential operator sites, a 10 bp inverted repeat (light blue) and an 18 bp direct repeat (dark blue), were identified in the Shal_3129-regulated promoter ($P_{phsA342}$). Each 18 bp repeat contains a 6 bp inverted repeat (black) separated by 2 bp, and therefore may be a complete operator. A consensus CRP binding site (purple) is located between the two 18 bp repeats. Truncation sites are indicated with arrows and the position in the promoter sequence. **(B)** Response of promoter truncations to thiosulfate. White bars are sfGFP fluorescence in the absence of ligand, black bars are with 5 mM thiosulfate, and red circles represent the fold induction (sfGFP fluorescence 5 mM/0 mM). Deletion of the first 150 bp of the 342 bp intergenic region ($P_{phsA151\text{-}342}$) had no effect on thiosulfate-induced promoter activation, indicating the 10 bp inverted repeat is not the ThsR binding site. Removal of the first 18 bp direct repeat (dark blue) also had no effect on promoter activation. Deletion of the promoter through the predicted CRP site or mutagenesis of the CRP site diminished sensor output ($P_{phsA182\text{-}342}$, $P_{phsA342\sim CRP}$; 25-fold to 4-fold), and deletion of the second 18 bp repeat abolished thiosulfate-induced signaling ($P_{phsA219\text{-}342}$).

Mutagenesis of either 6 bp inverted repeat in the second 18 bp element ($P_{PhsA342\sim O1}$, $P_{PhsA342\sim O2}$) also resulted in loss of signaling, supporting this sequence as the likely operator site. Mutated sequences are indicated under the wild type promoter sequence. No truncations were identified that gave enhanced promoter response, therefore the entire intergenic region was used as the output promoter.

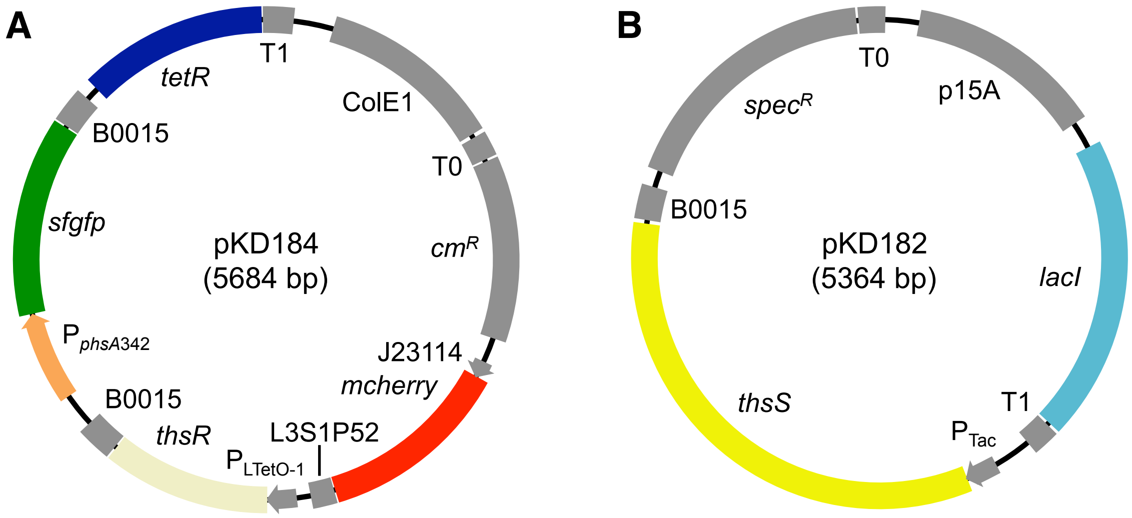

Appendix Figure S3. Plasmid maps for inducible ThsSR production.

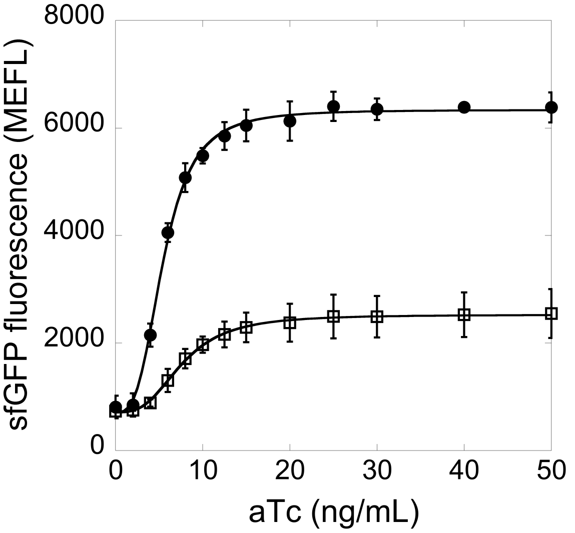

Appendix Figure S4. Shal_3129-induced activation of $P_{\mathit{phsA}342}$. Closed circles represent the wild type and open squares the phospho-accepting null mutant (D57A).

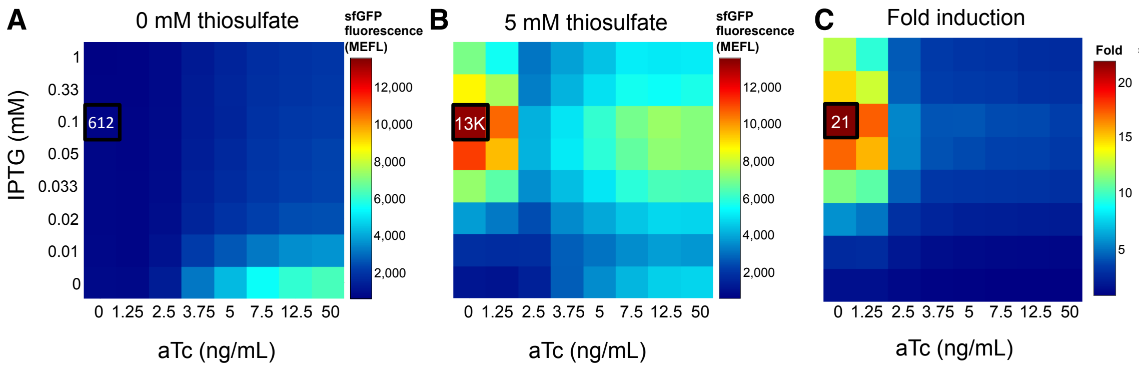

Appendix Figure S5. Optimization of protein expression using inducible promoters to give the highest dynamic for ThsSR. Data are shown for all aTc and IPTG conditions tested in the **(A)** absence of thiosulfate (0 mM), **(B)** presence of saturating thiosulfate (5 mM), and **(C)** the fold difference between the two (sfGFP fluorescence 5 mM/0 mM thiosulfate).

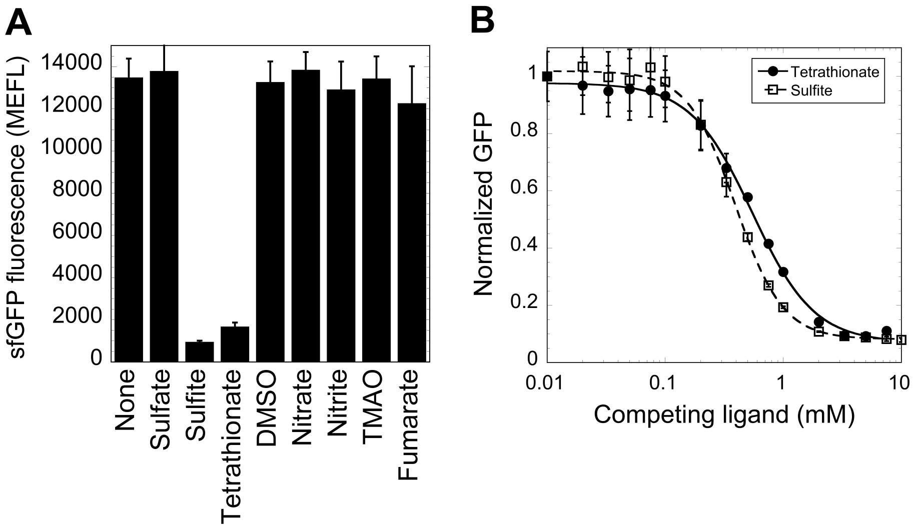

Appendix Figure S6. Ligand inhibition assay for ThsSR. **(A)** ThsSR response in presence of 5 mM thiosulfate and 10 mM other TEAs. A decrease in response indicates inhibition by the added ligand. **(B)** Inhibition curve of tetrathionate (filled circles) and sulfite (open squares) in the presence of 5 mM thiosulfate.

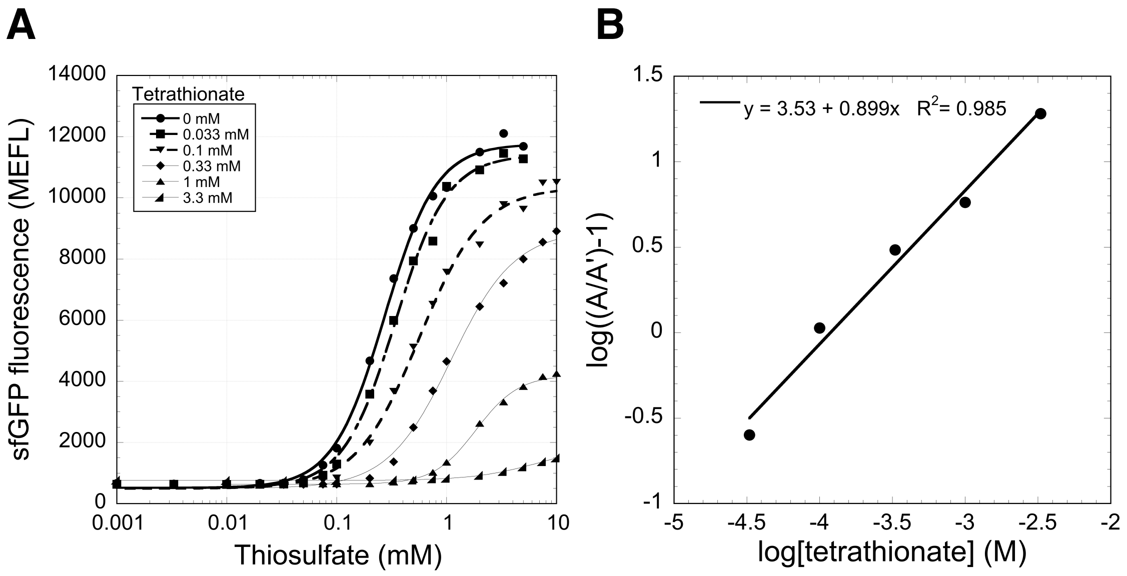

Appendix Figure S7. Schild plot analysis of ThsSR tetrathionate inhibition. **(A)** An increase in $k_{1/2}$ of the sensor was observed at higher concentrations of tetrathionate, however maximal response could not be achieved in the presence of tetrathionate. Thiosulfate concentrations >10 mM were not used because of an additional low-sensitivity response of ThsSR that would complicate analysis. **(B)** Schild plot of dose-response data. Slope of the Schild plot was not 1 indicating tetrathionate is not a competitive antagonist or that the system is not in equilibrium.

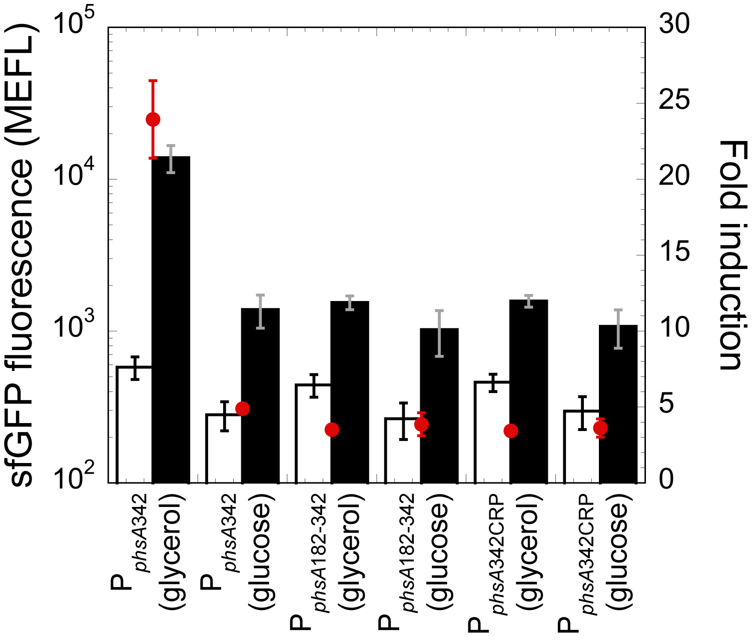

Appendix Figure S8. Glucose sensitivity of thiosulfate-induced activation of ThsSR at P_{phsA}. The full-length promoter ($P_{phsA342}$), a 5' truncated promoter removing the CRP site ($P_{phsA182-342}$), and a promoter with CRP binding motif mutated ($P_{phsA342CRP}$) were tested in M9 minimal media with 0.4% glycerol or 0.4% glucose as the carbon source. White bars are in the absence of thiosulfate, black bars are in the presence of 5 mM thiosulfate, and red circles are the fold induction (sfGFP fluorescence 5 mM/0 mM).

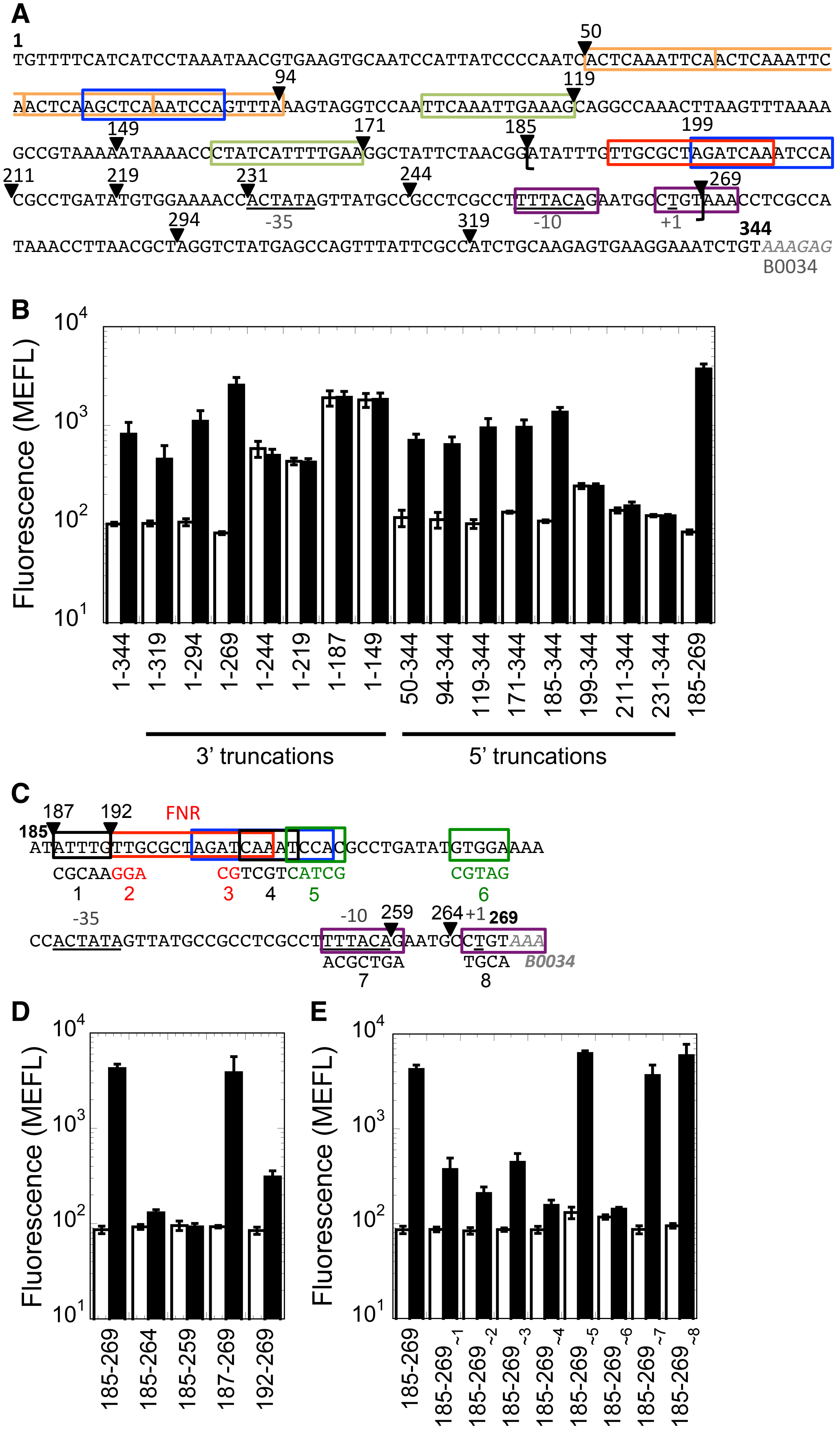

A
B
Fluorescence (MEFL)
3' truncations
5' truncations
C
FNR
D
E
B0034

Appendix Figure S9. Identification of $P_{ttrB344}$ regulatory features. **(A)** Potential operator sites based on direct or inverted repeat sequences are highlighted in a variety of colors. Truncation sites are indicated with arrows and the position in the promoter sequence. The predicted -35, -10 and +1 sites are based on sequence similarity to P_{ttrB} from *S. typhimurium.* **(B)** Response of P_{ttrB} promoter truncations to tetrathionate. The numbers indicated in the x-axis are the bps in the truncated sequence relative to the original 344 bp intergenic region. White bars are fluorescence in the absence of ligand and black bars are in the presence of 1 mM tetrathionate. Deletion of the first 184 bp of the 344 bp intergenic region ($P_{ttrB185\text{-}344}$) had no effect on thiosulfate-induced promoter activation, indicating the majority of the highlighted sequences are not the operator site. Removal of the terminal 75 bp ($P_{ttrB1\text{-}269}$) resulted in decreased basal fluorescence in the absence of tetrathionate and increased fluorescence in the presence of tetrathionate, relative to the full intergenic region. A minimal promoter with these combined truncations ($P_{ttrB185\text{-}269}$) gave improved performance over the full-length promoter sequence. This truncated promoter also minimizes the potential for cross-talk from other transcription factors and was therefore used as the TtrSR output promoter in this study. **(C)** Additional promoter characterization of the minimal promoter $P_{ttrB185\text{-}269}$. Potential FixJ-like operator half sites are highlighted in black, green, and purple, and the FNR site in red. Promoter mutations are indicated below the wild type sequence and truncation positions are indicated with arrows. **(D)** P_{ttrB} could be further truncated at the 5' end by two bp to $P_{ttrB187\text{-}269}$ with no loss of function, however removal of the first inverted repeat highlighted in black decreased promoter output 14-fold ($P_{ttrB192\text{-}269}$). No further truncation was tolerated at the 3' end, likely due to removal of the +1 transcriptional start site. **(E)** Mutagenesis of regions of interest within the truncated promoter $P_{ttrB185\text{-}269}$.

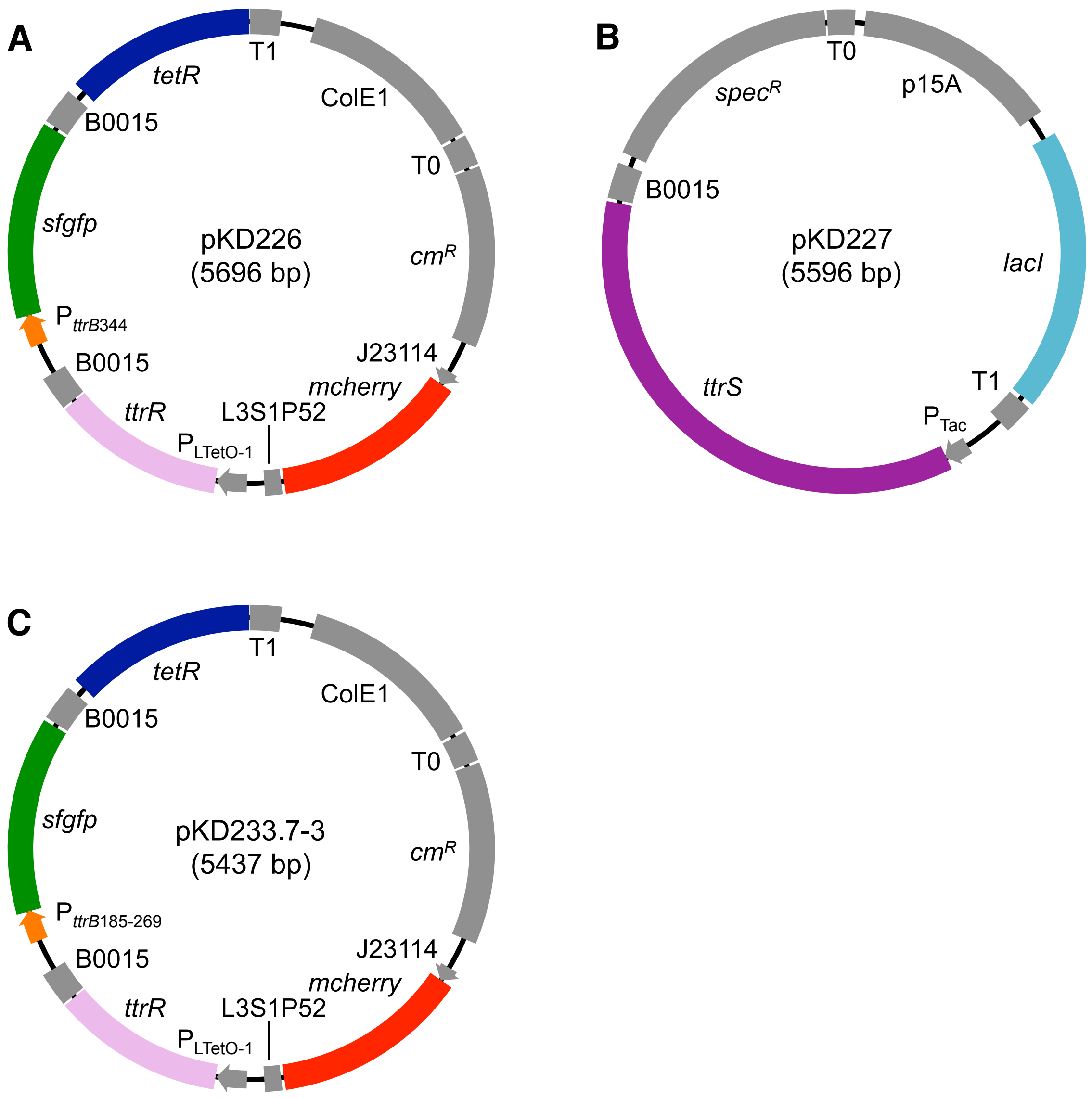

Appendix Figure S10. Plasmid maps for inducible TtrSR production.

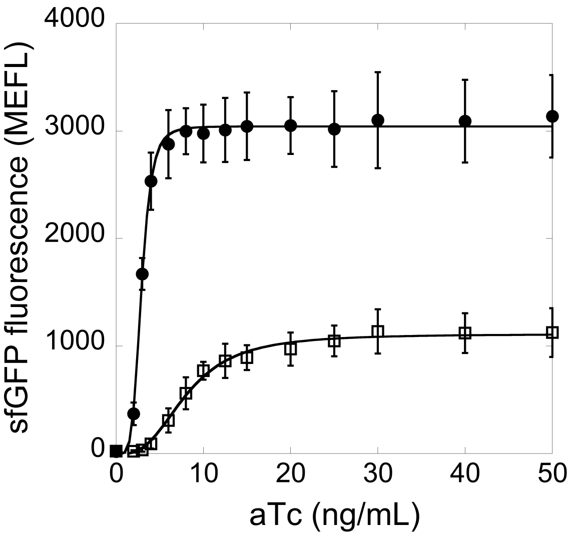

Appendix Figure S11. Sbal195_3858-induced activation of $P_{TtrB344}$. Closed circles represent the wild type and open squares the phospho-accepting null mutant (D55A).

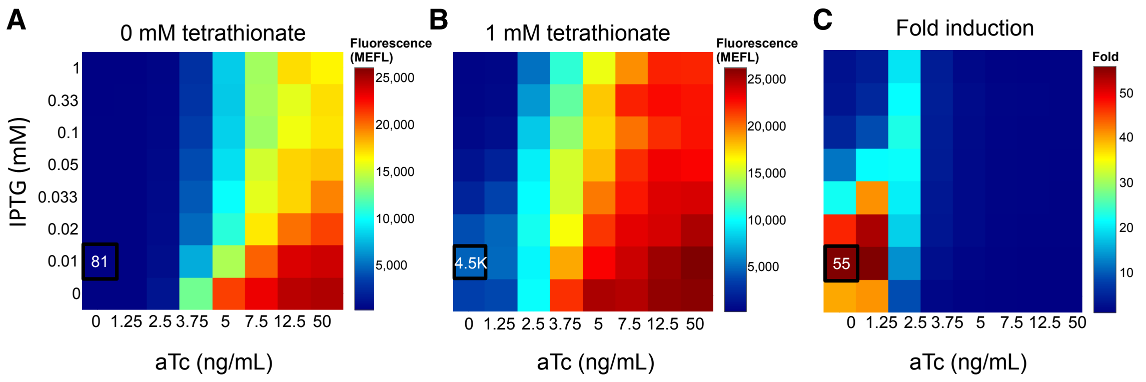

Appendix Figure S12. Optimization of protein expression using inducible promoters to give the highest dynamic for TtrSR. Data are shown for all aTc and IPTG conditions tested in the **(A)** absence of tetrathionate (0 mM), **(B)** presence of saturating tetrathionate (1 mM), and **(C)** the fold difference between the two (fluorescence 1 mM/0 mM tetrathionate).

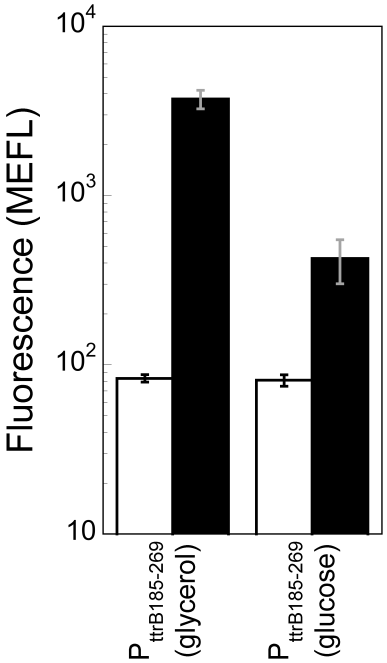

Appendix Figure S13. Glucose sensitivity of tetrathionate-induced activation of $P_{\mathit{ttrB}185\text{-}269}$. White bars are with 0 mM tetrathionate and black bars with 1 mM tetrathionate.

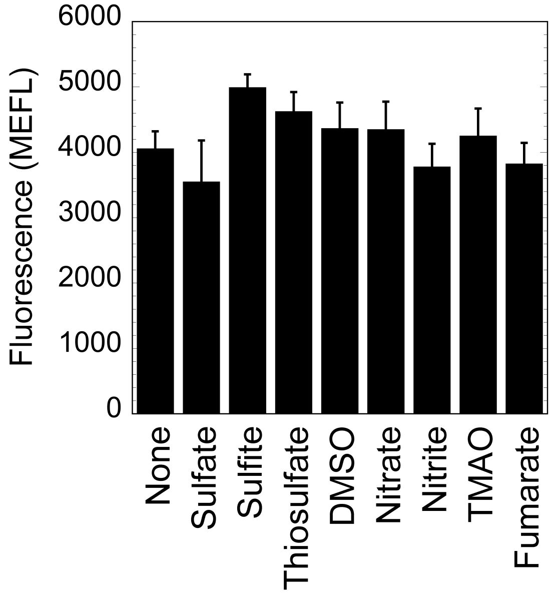

Appendix Figure S14. TtrSR response in presence of 1 mM tetrathionate and 10 mM other TEAs.

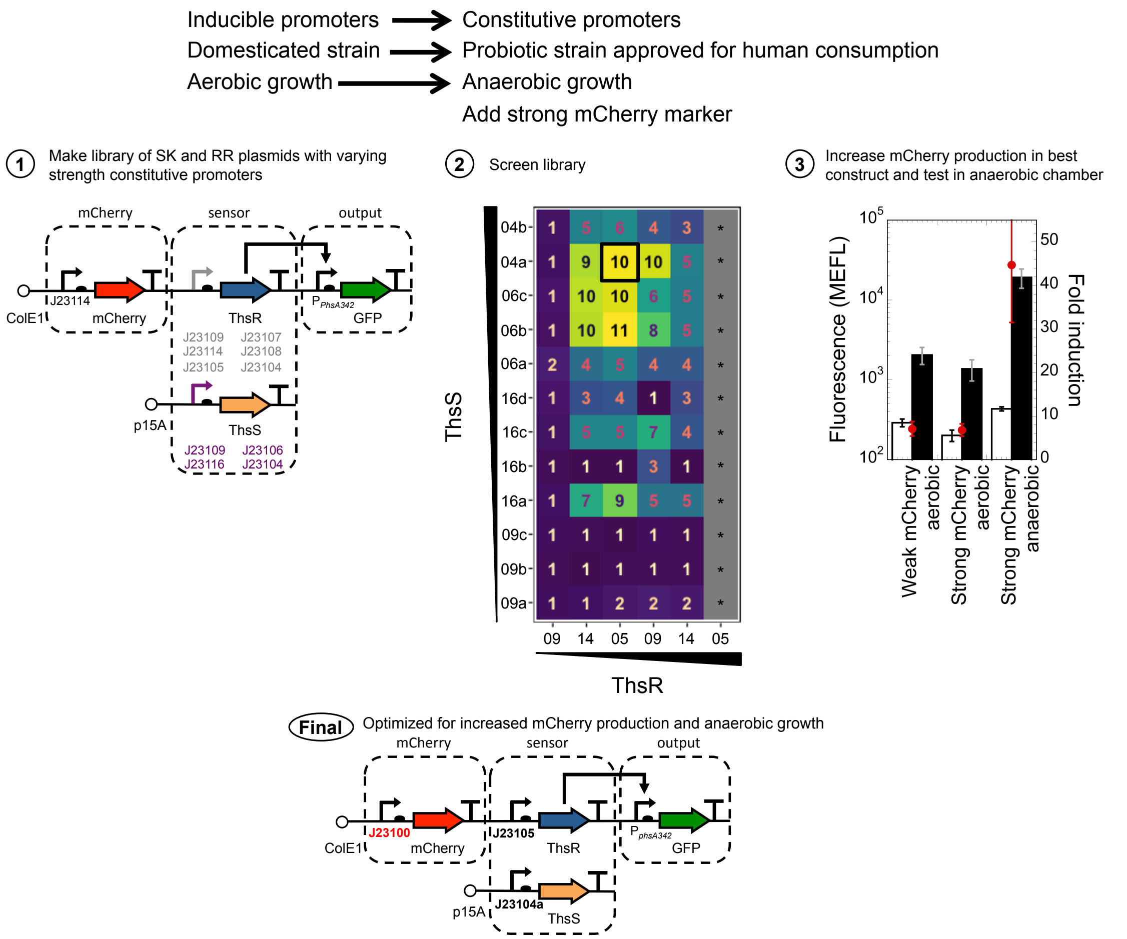

Appendix Figure S15. Optimization of the ThsSR for use in the mammalian gut. **1)** A library of varying strength constitutive promoters from the Anderson promoter library (indicated by identity J231XX) and designed synthetic ribosome binding sites (RBS) (if multiple were designed per promoter, they are designated with the letters a-d) were incorporated upstream of ThsS and ThsR. **2)** The ThsR and ThsS plasmids were then combined together and screened for optimal expression in *E. coli* Nissle 1917 (fold induction +/- 5 mM thiosulfate is indicated). Combinations that had significant growth defects are indicated in grey with a *. **3)** In the best construct, the promoter of the constitutive mCherry marker was increased to allow for selection of the biosensor from the normal microbial floral and the construct was tested in anaerobic growth conditions. The final constructs used for *in vivo* studies are shown at the bottom.

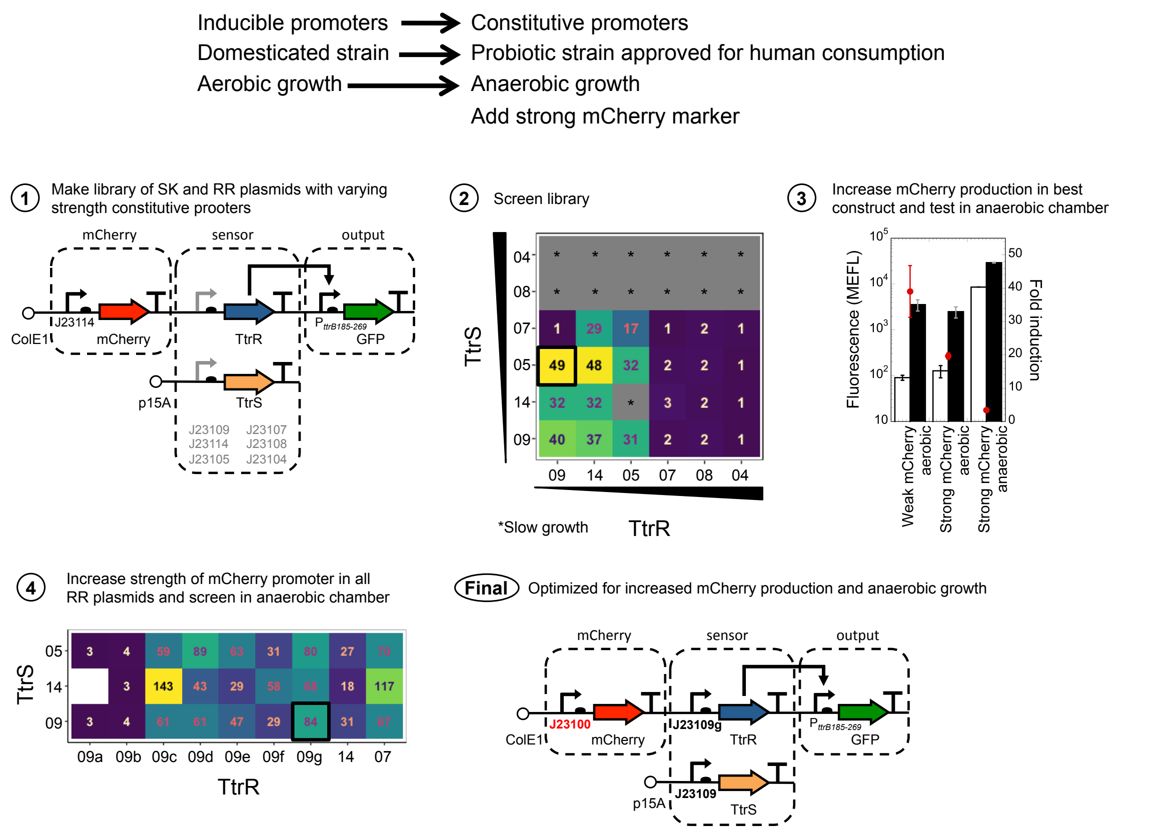

Appendix Figure S16. Optimization of TtrSR for use in the mammalian gut. **1)** A library of varying strength constitutive promoters from the Anderson promoter library (indicated by identity J231XX) and a designed synthetic RBS were incorporated upstream of TtrS and TtrR. **2)** The TtrR and TtrS plasmids were then combined together and screened for optimal expression in *E. coli* Nissle 1917 (fold induction +/- 1 mM tetrathionate is indicated). Combinations that had significant growth defects are indicated in grey with a *. **3)** In the best construct, the promoter of the constitutive mCherry marker was increased to allow for selection of the biosensor from the normal microbial floral and the construct was tested in anaerobic growth conditions. This resulted in a significant decrease in sensor performance. Because the best construct from the initial screen was the weakest promoter, additional synthetic RBSs designed to be weaker were designed with this promoter and the strong mCherry promoter was incorporated into all RR plasmids. These plasmids were then combined and screened in anaerobic conditions. Although the best construct appears to be TtrR 09c and TtrS 14, the inactivating control plasmid (D55A) was not made in time for in vivo experiments. Therefore, the best available plasmid was used (indicated by a black box) which gave good dynamic range. The final constructs used for *in vivo* studies are shown at the bottom.

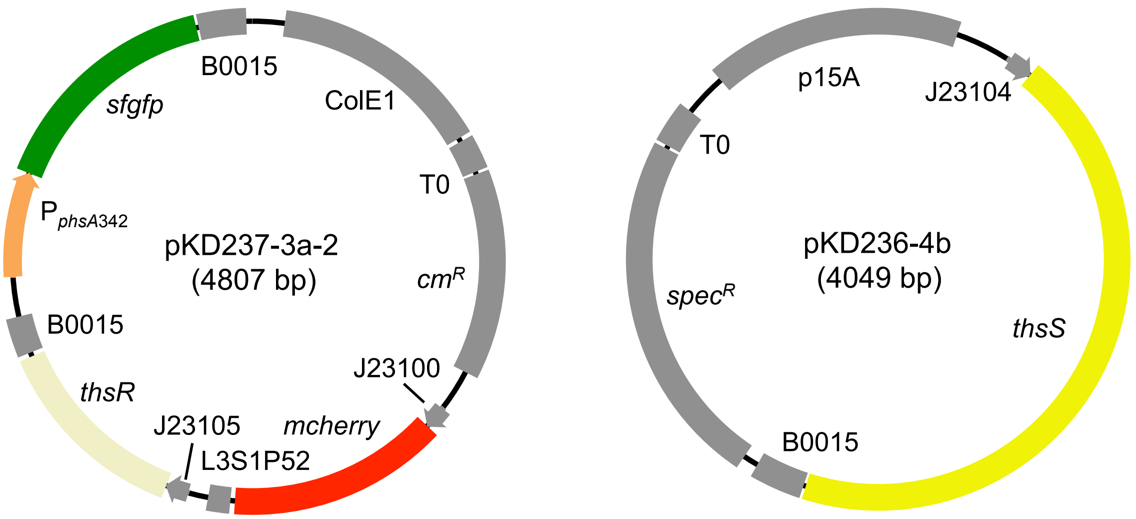

Appendix Figure S17. Plasmid maps for constitutive ThsSR production used in *in* vivo studies.

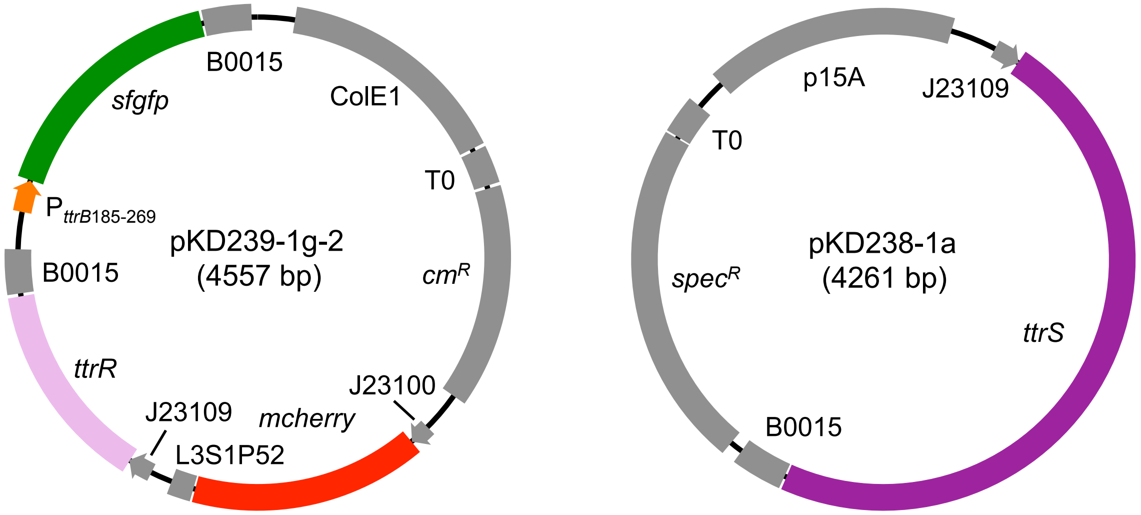

Appendix Figure S18. Plasmid maps for constitutive TtrSR production used in *in* vivo studies.

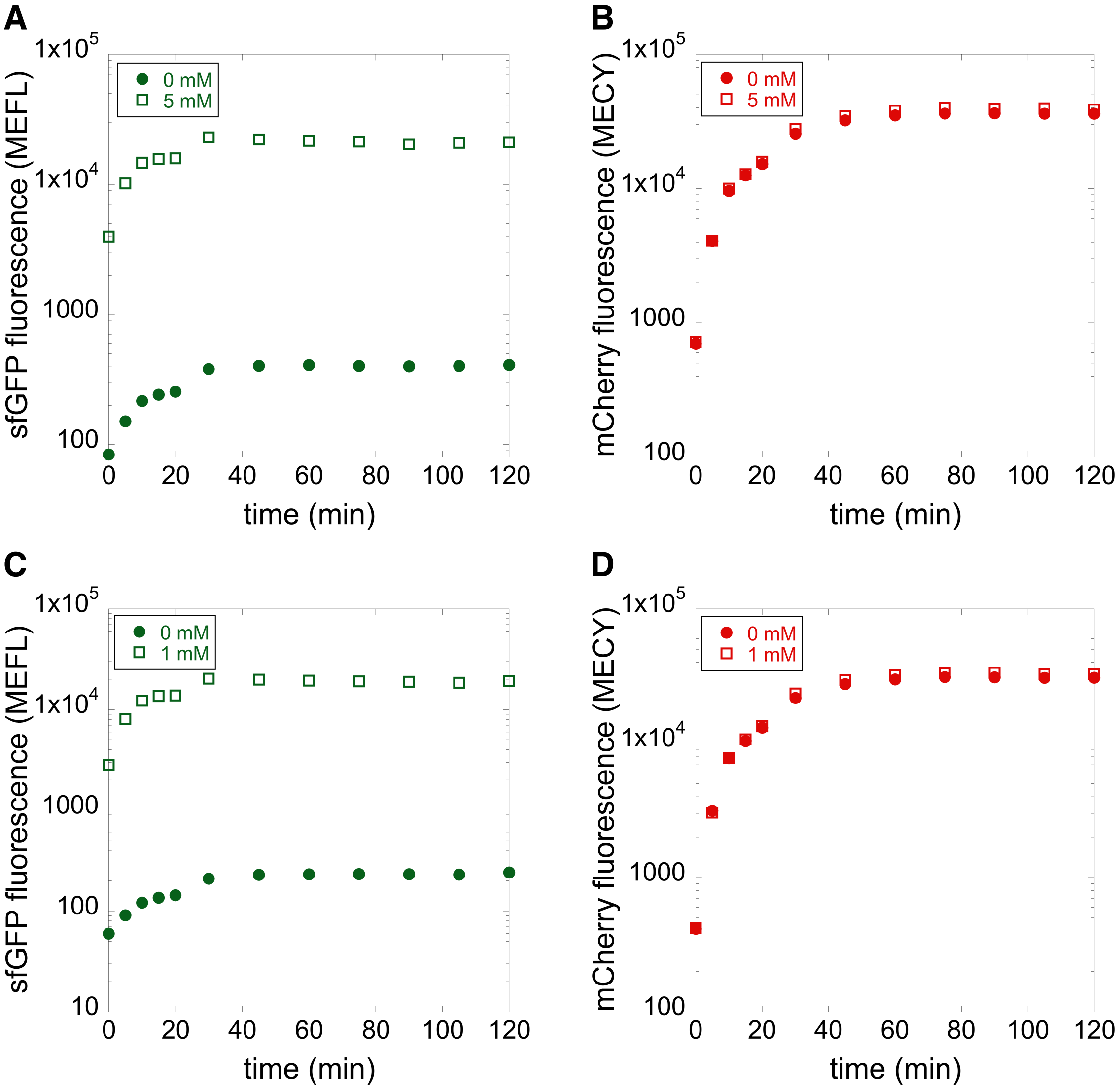

Appendix Figure S19. Fluorophore maturation time course of anaerobically grown Nissle bacteria in PBS+1 mg/mL chloramphenicol. (**A**) sfGFP and (**B**) mCherry fluorescence over time for ThsSR in the presence and absence of saturating thiosulfate. (**C**) sfGFP and (**D**) mCherry fluorescence over time for *S. baltica* TtrSR in the presence and absence of saturating tetrathionate.

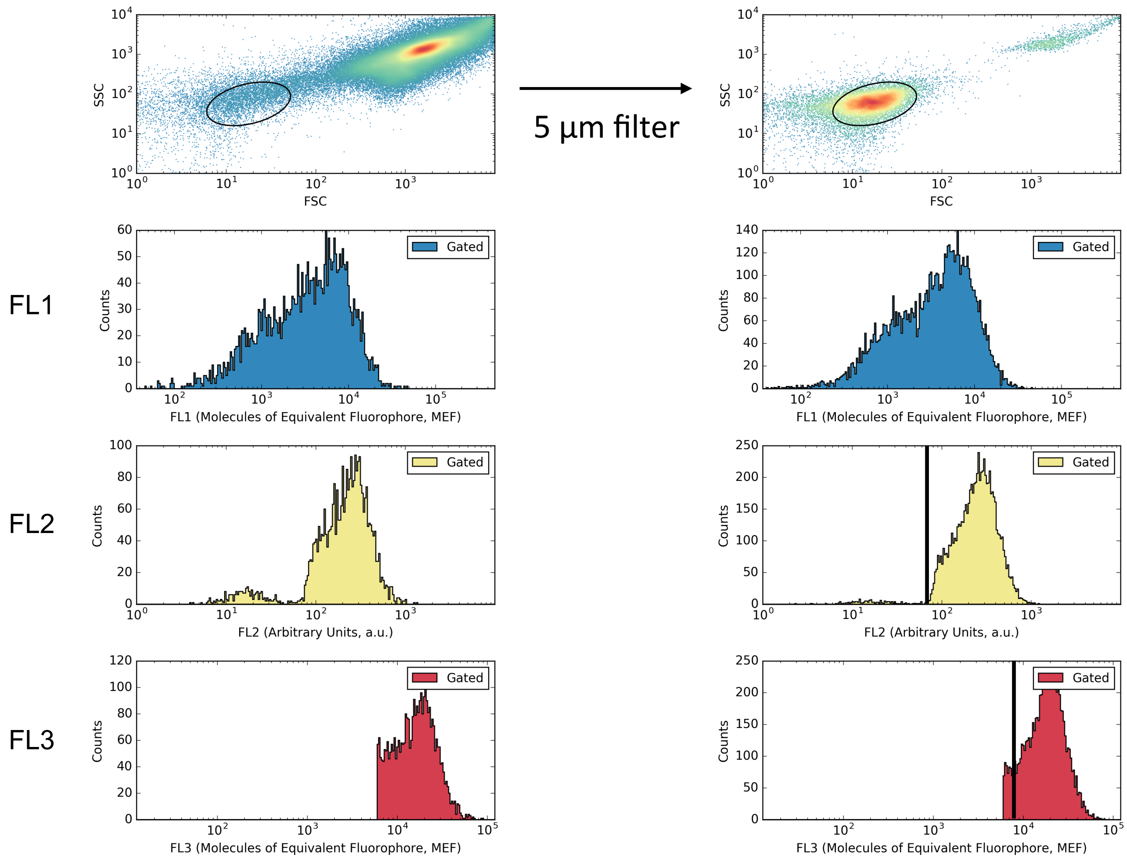

Appendix Figure S20. Flow cytometry profile before and after filtering through a 5 µM syringe filter. Data are only collected for cytometer counts demonstrating high mCherry fluorescence on the FL3 channel. The majority of counts from fecal and colon samples fall below this threshold and are not recorded by the cytometer. Data are then gated to only include counts with a similar FSC and SSC profile of *E. coli* (black ellipse), and with high fluorescence in both the FL2 and FL3 channels (to the right of the black bar).

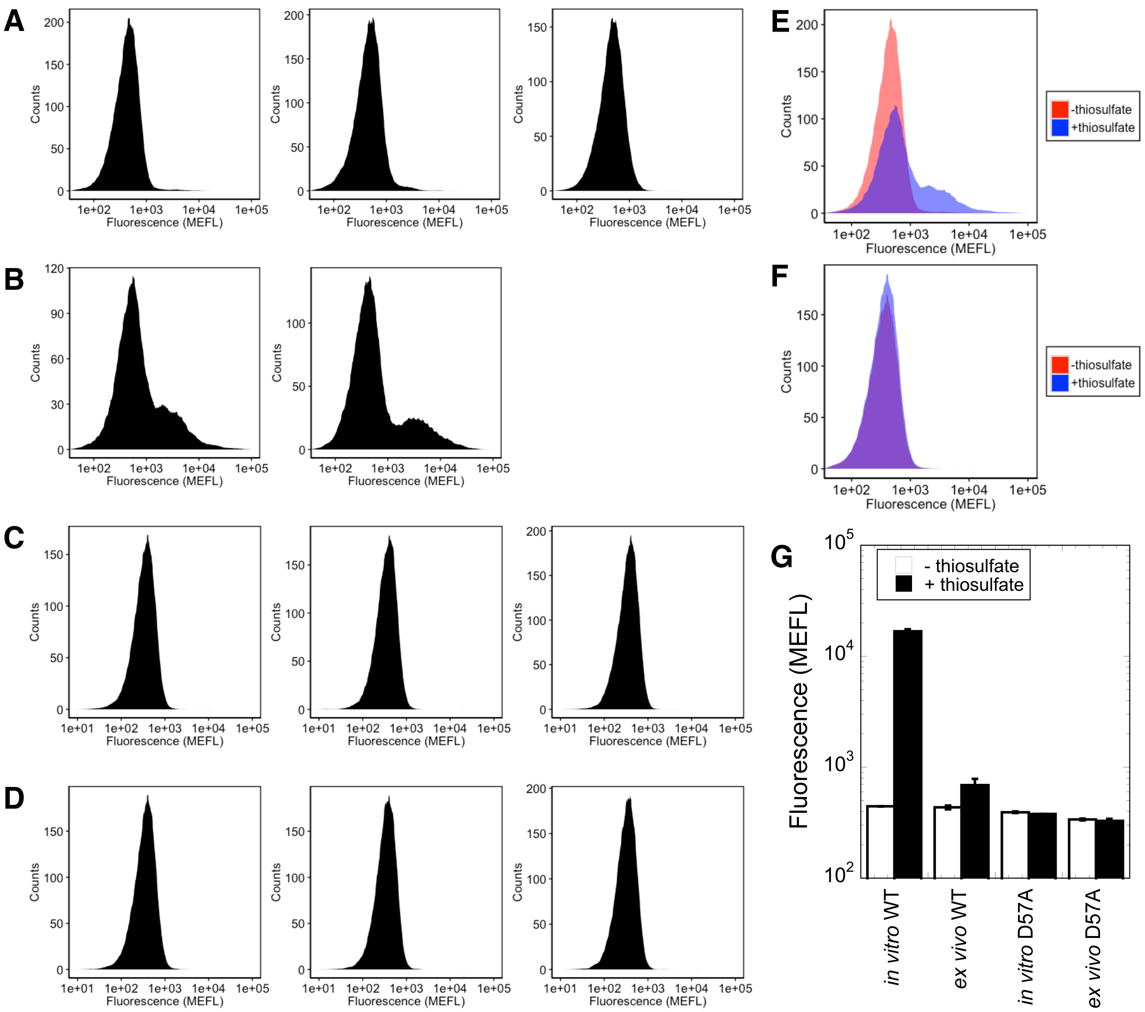

Appendix Figure S21. ThsSR performance in *ex vivo* ligated colon explants. ThsSR response after 6 hours incubation in the colon with **(A)** no added thiosulfate and **(B)** 5 mM added thiosulfate. Data shown are histograms of fluorescence from each colon (n=3). One colon from **(B)** was accidentally contaminated with tetrathionate and is therefore omitted from analysis. Inactivating control (D57A) incubated with **(C)** 0 mM thiosulfate and **(D)** 5 mM thiosulfate. Overlay histogram of one mouse colon from each tested condition: **(E)** ThsSR (-/+ thiosulfate) and **(F)** ThsSR D57A (-/+ thiosulfate). **(G)** Comparison of ThsSR performance *in vitro* and *ex vivo*. Data is mean +/- s.d. The histograms of the data are shown because of the small sample size of the experiment, which prevents effective determination of normality. Assuming equal variance, the p-values are 0.017 – vs. + thiosulfate (*ex vivo* WT) and 0.0061 for *ex vivo* WT vs. D57A (+ thiosulfate). Assuming unequal variance, the p-values are 0.16 and 0.12, respectively.

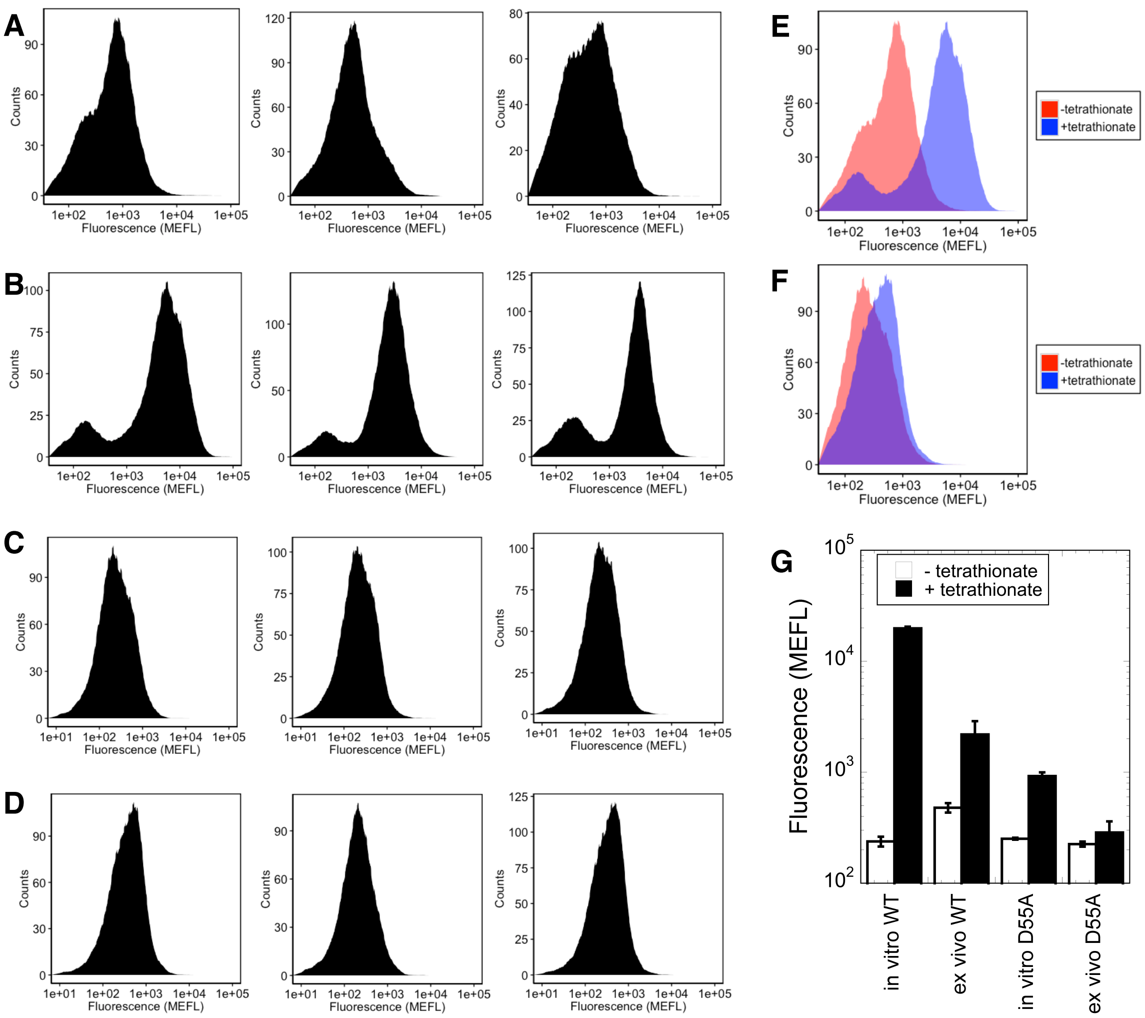

Appendix Figure S22. *S. baltica* TtrSR performance in *ex vivo* ligated colon explants. TtrSR response after 6 hours incubation in the colon with **(A)** no added tetrathionate and **(B)** 1 mM added tetrathionate. Data shown are histograms of fluorescence from each colon (n=3). Inactivating control (D55A) incubated with **(C)** 0 mM tetrathionate and **(D)** 1 mM tetrathionate. Overlay histogram of one mouse colon from each tested condition: **(E)** TtrSR (-/+ tetrathionate) and **(F)** TtrSR D55A (-/+ tetrathionate). **(G)** Comparison of TtrSR performance *in vitro* and *ex vivo*. Data is mean +/- s.d.

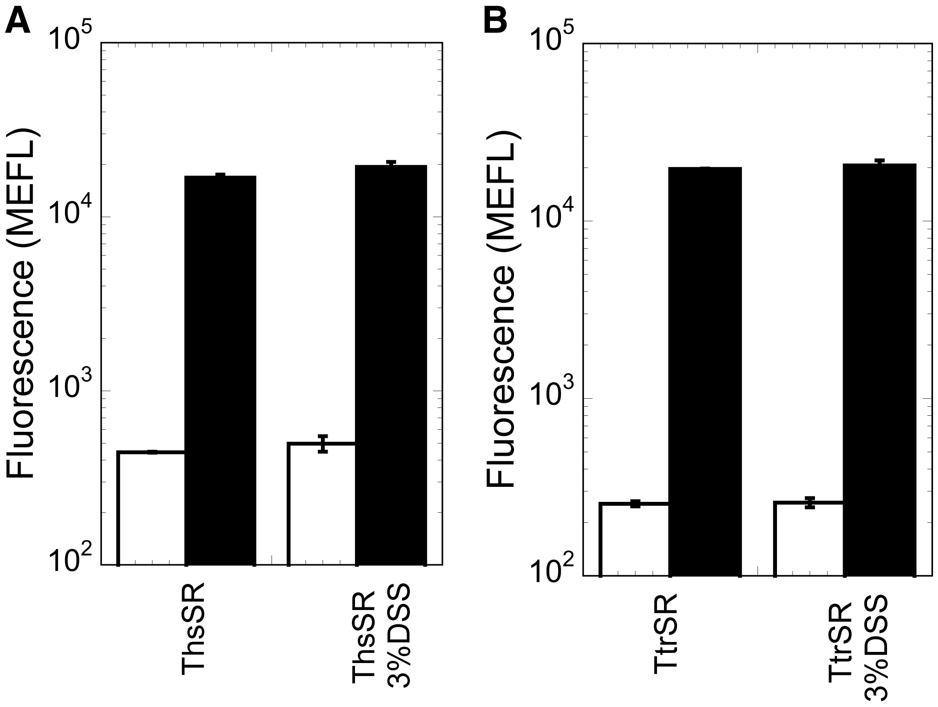

Appendix Figure S23. **(A)** ThsSR and **(B)** TtrSR sensor performance with and without 3% DSS. White bars are with no ligand and black bars are with saturating ligand (5 mM thiosulfate and 1 mM tetrathionate, respectively).

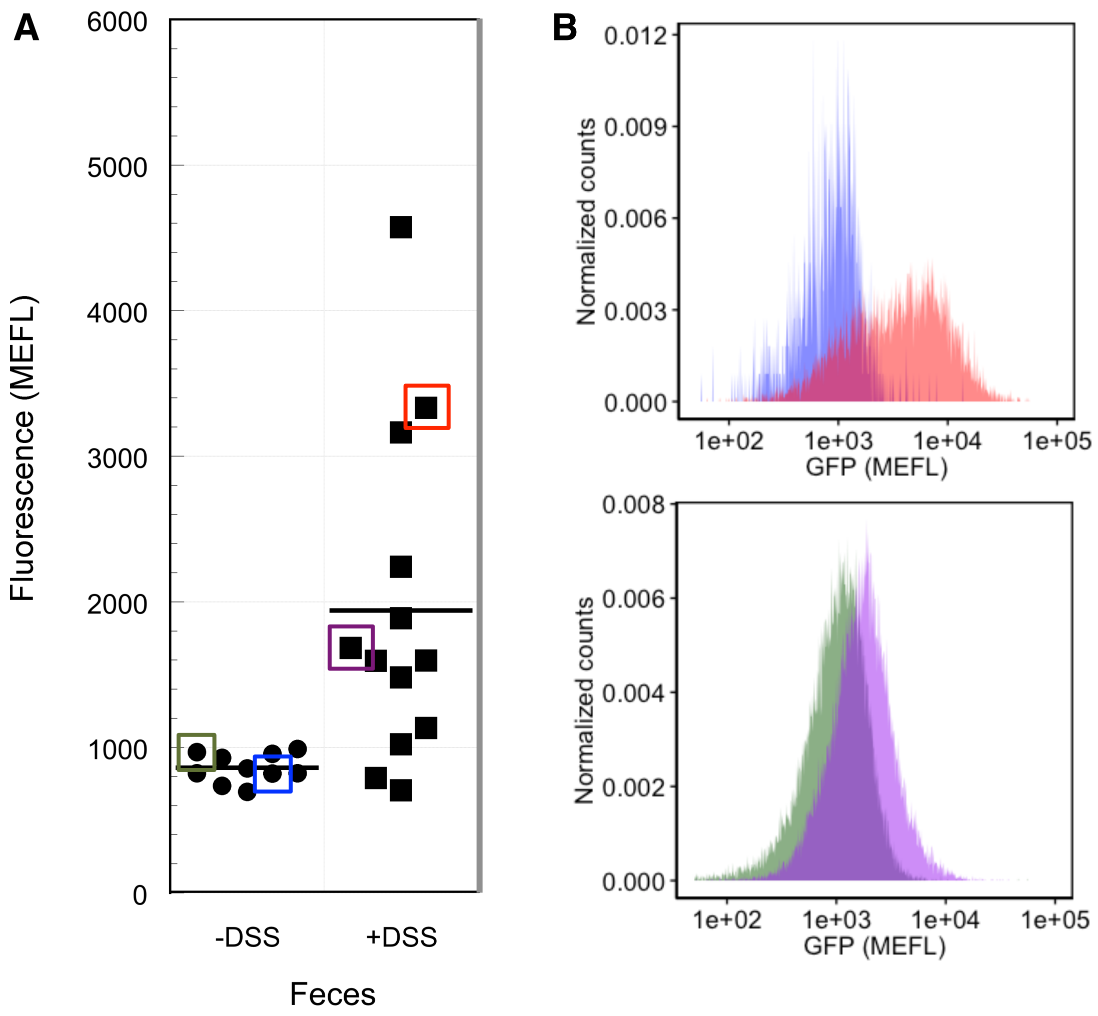

Appendix Figure S24. Histograms of representative fecal samples from healthy control mice (green and blue) and DSS-treated mice (purple and red).

A

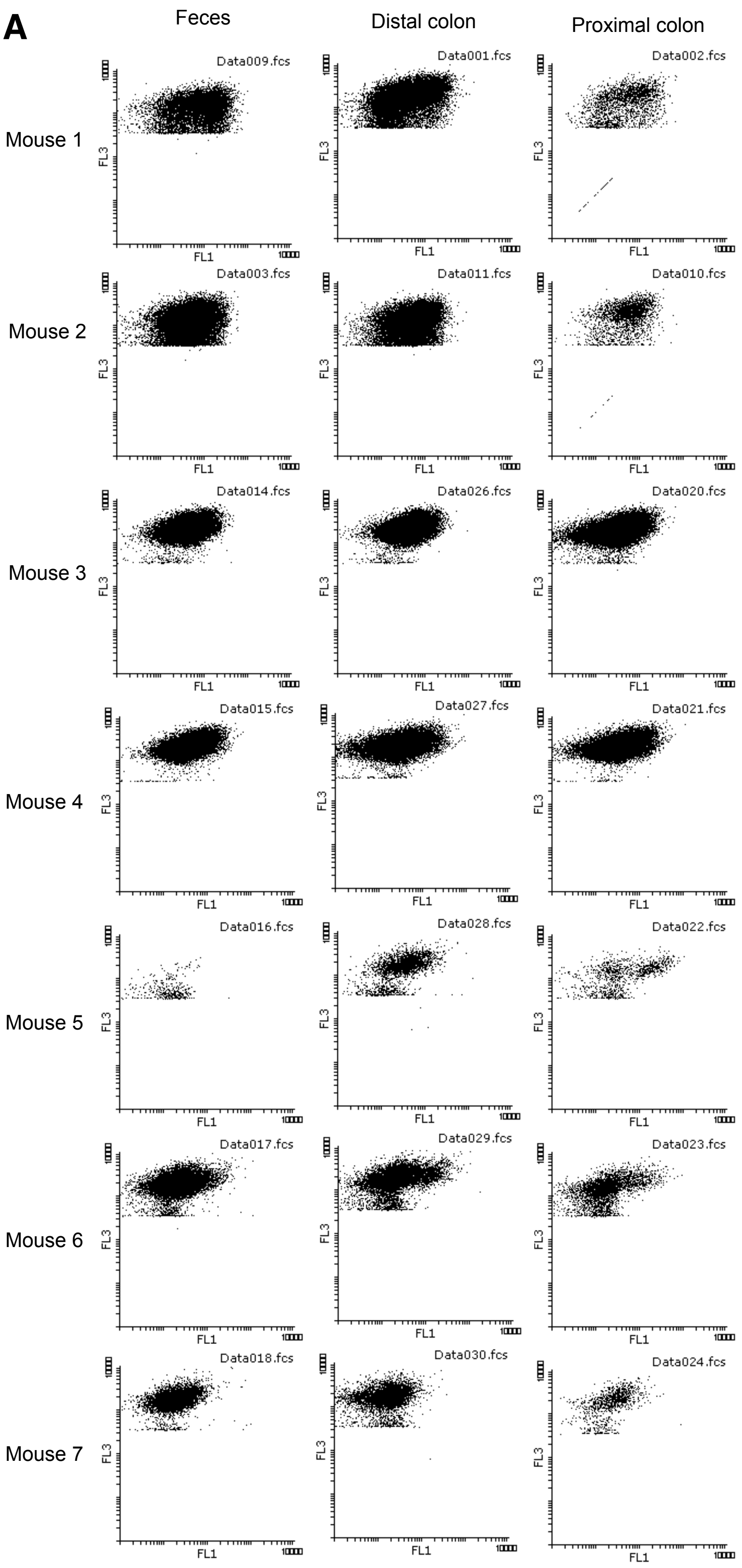
Feces
Distal colon
Proximal colon
Mouse 1
Mouse 2
Mouse 3
Mouse 4
Mouse 5
Mouse 6
Mouse 7
Data009.fcs
Data001.fcs
Data002.fcs
Data003.fcs
Data011.fcs
Data010.fcs
Data014.fcs
Data026.fcs
Data020.fcs
Data015.fcs
Data027.fcs
Data021.fcs
Data016.fcs
Data028.fcs
Data022.fcs
Data017.fcs
Data029.fcs
Data023.fcs
Data018.fcs
Data030.fcs
Data024.fcs
FL1
FL3

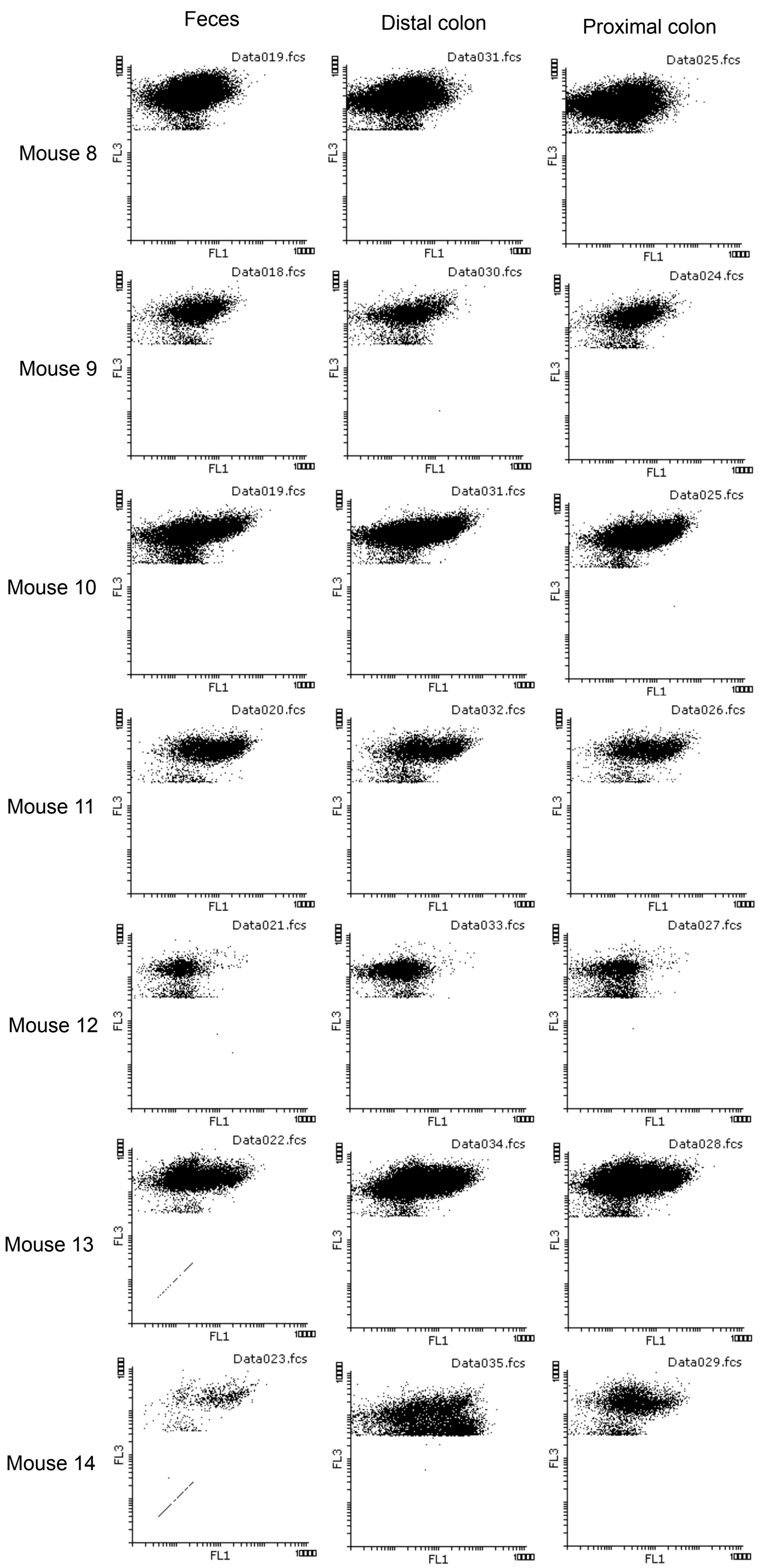

Feces
Distal colon
Proximal colon
Mouse 8
Mouse 9
Mouse 10
Mouse 11
Mouse 12
Mouse 13
Mouse 14
Data019.fcs
Data031.fcs
Data025.fcs
Data018.fcs
Data030.fcs
Data024.fcs
Data019.fcs
Data031.fcs
Data025.fcs
Data020.fcs
Data032.fcs
Data026.fcs
Data021.fcs
Data033.fcs
Data027.fcs
Data022.fcs
Data034.fcs
Data028.fcs
Data023.fcs
Data035.fcs
Data029.fcs
FL1
FL3

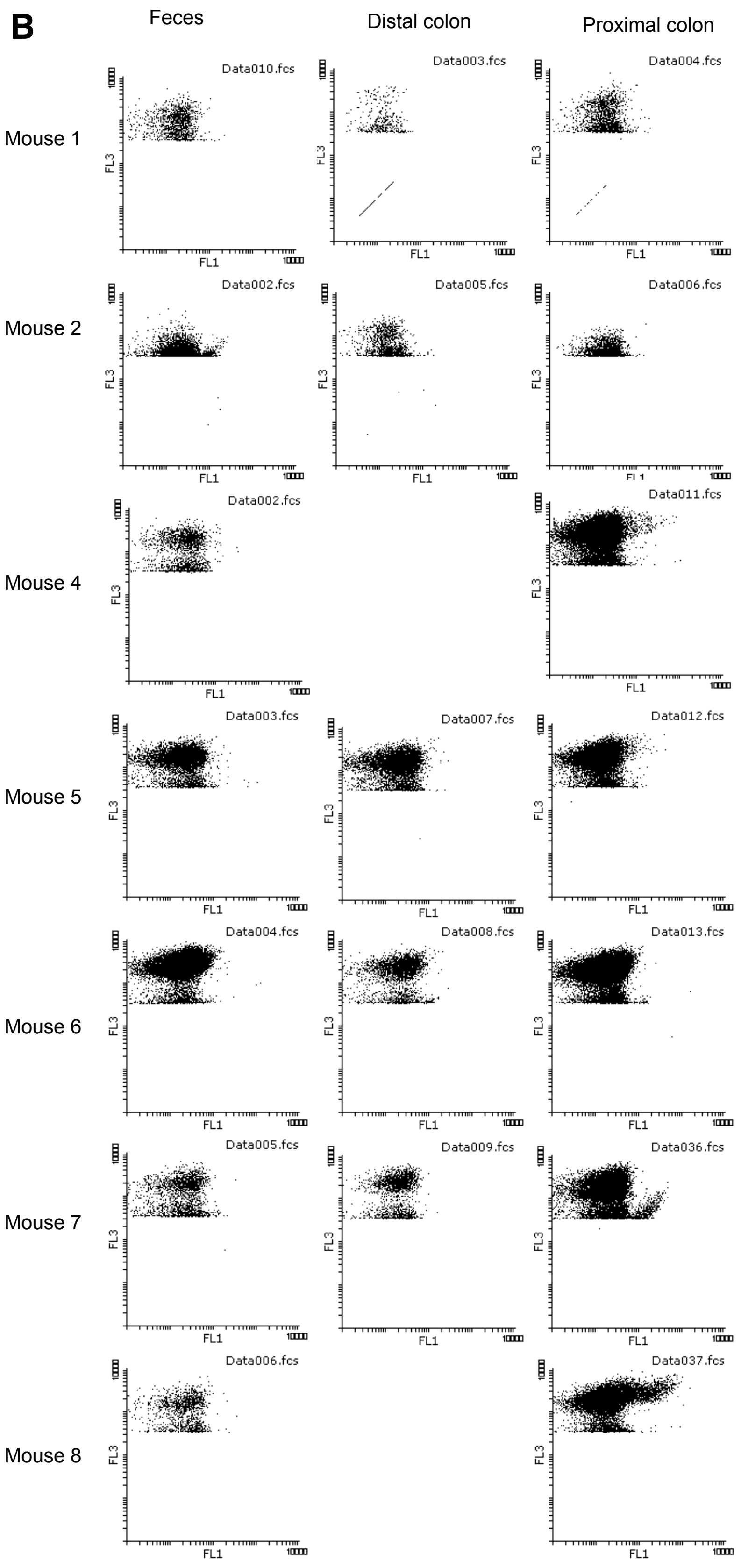

B
Feces
Distal colon
Proximal colon
Mouse 1
Mouse 2
Mouse 4
Mouse 5
Mouse 6
Mouse 7
Mouse 8
Data010.fcs
Data003.fcs
Data004.fcs
Data002.fcs
Data005.fcs
Data006.fcs
Data002.fcs
Data011.fcs
Data003.fcs
Data007.fcs
Data012.fcs
Data004.fcs
Data008.fcs
Data013.fcs
Data005.fcs
Data009.fcs
Data036.fcs
Data006.fcs
Data037.fcs
FL1
FL3

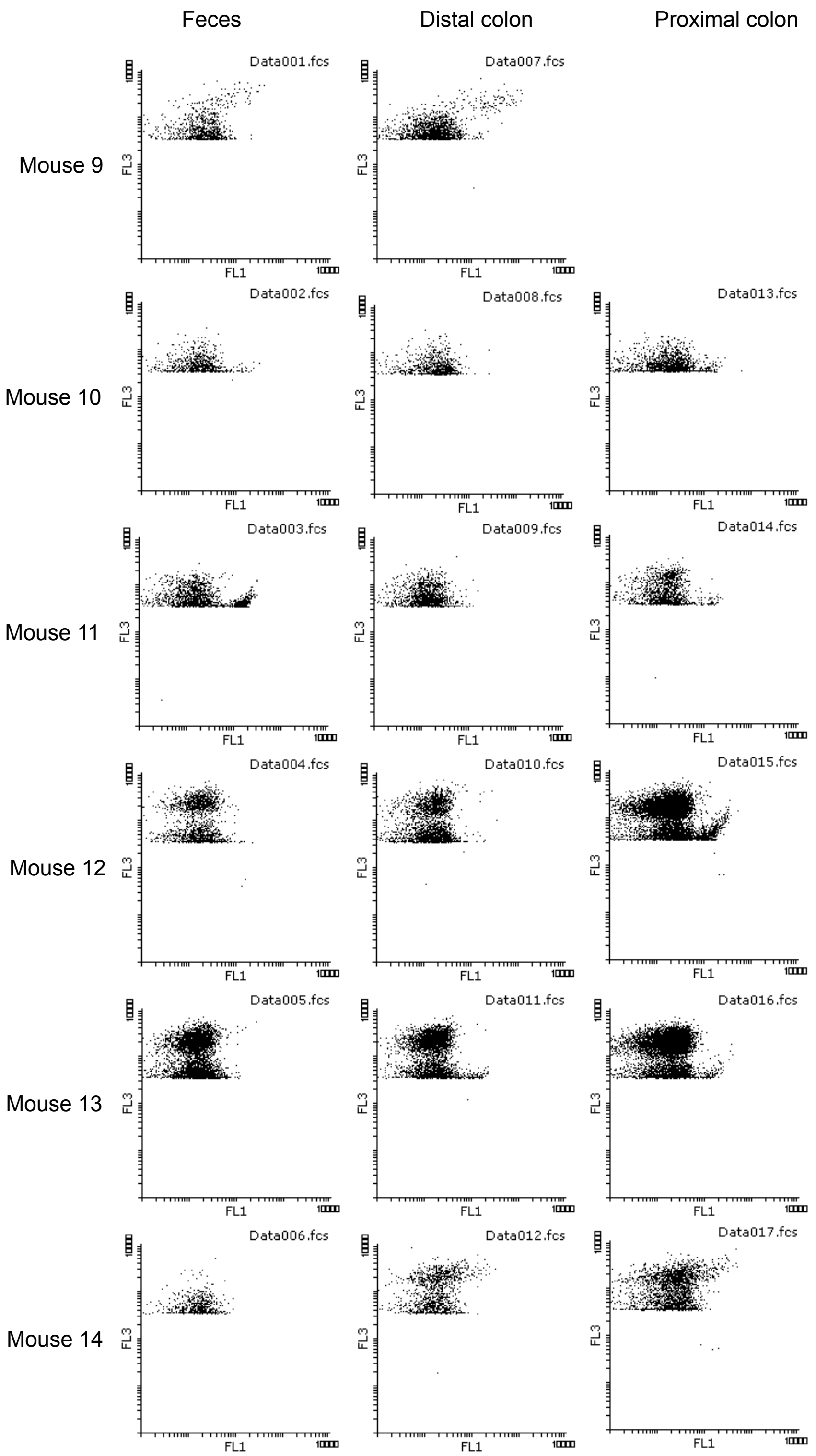

Feces
Distal colon
Proximal colon
Mouse 9
Mouse 10
Mouse 11
Mouse 12
Mouse 13
Mouse 14
Data001.fcs
Data002.fcs
Data003.fcs
Data004.fcs
Data005.fcs
Data006.fcs
Data007.fcs
Data008.fcs
Data009.fcs
Data010.fcs
Data011.fcs
Data012.fcs
Data013.fcs
Data014.fcs
Data015.fcs
Data016.fcs
Data017.fcs
FL1
FL3

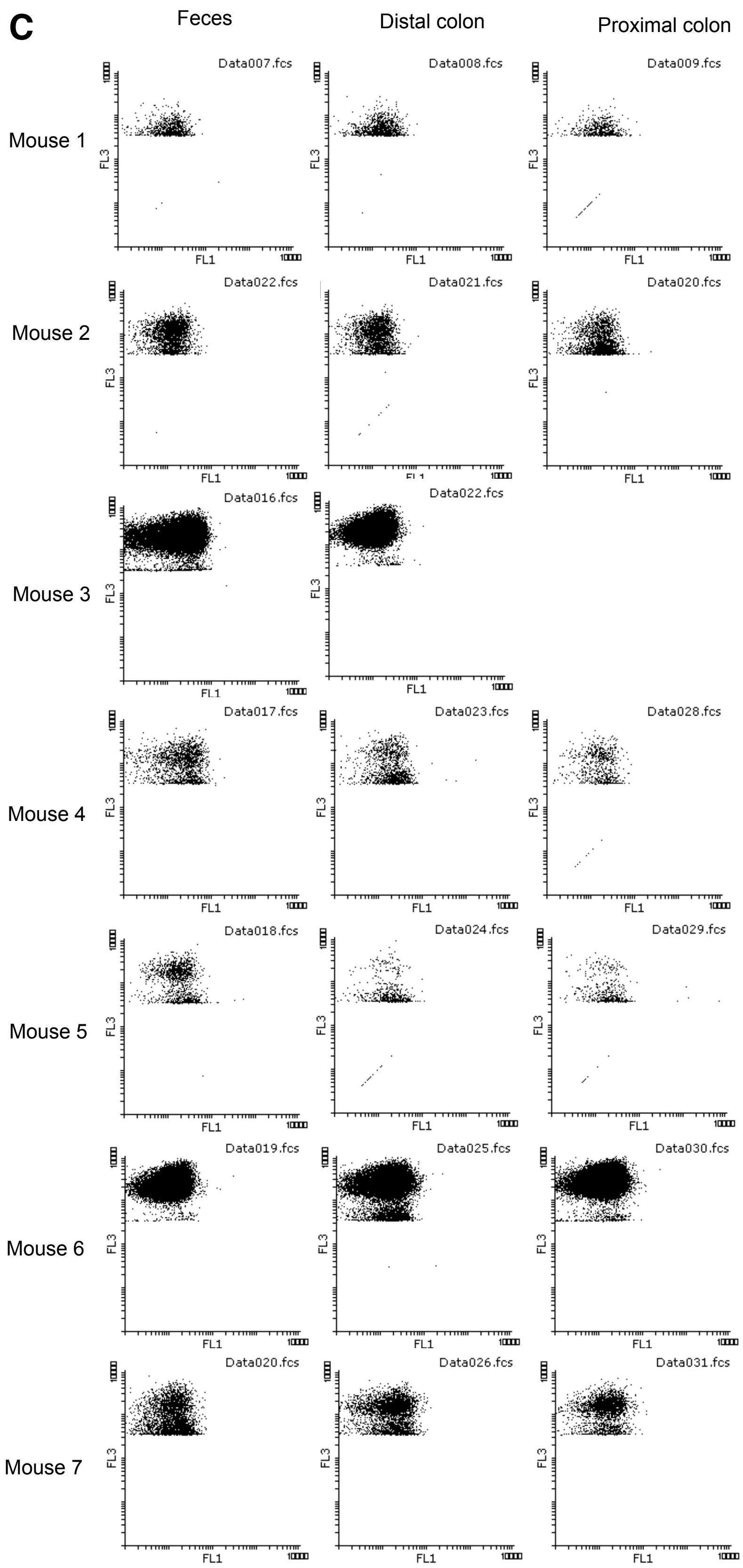
C
Feces
Distal colon
Proximal colon
Mouse 1
Data007.fcs
Data008.fcs
Data009.fcs
Mouse 2
Data022.fcs
Data021.fcs
Data020.fcs
Mouse 3
Data016.fcs
Data022.fcs
Mouse 4
Data017.fcs
Data023.fcs
Data028.fcs
Mouse 5
Data018.fcs
Data024.fcs
Data029.fcs
Mouse 6
Data019.fcs
Data025.fcs
Data030.fcs
Mouse 7
Data020.fcs
Data026.fcs
Data031.fcs
FL1
FL3

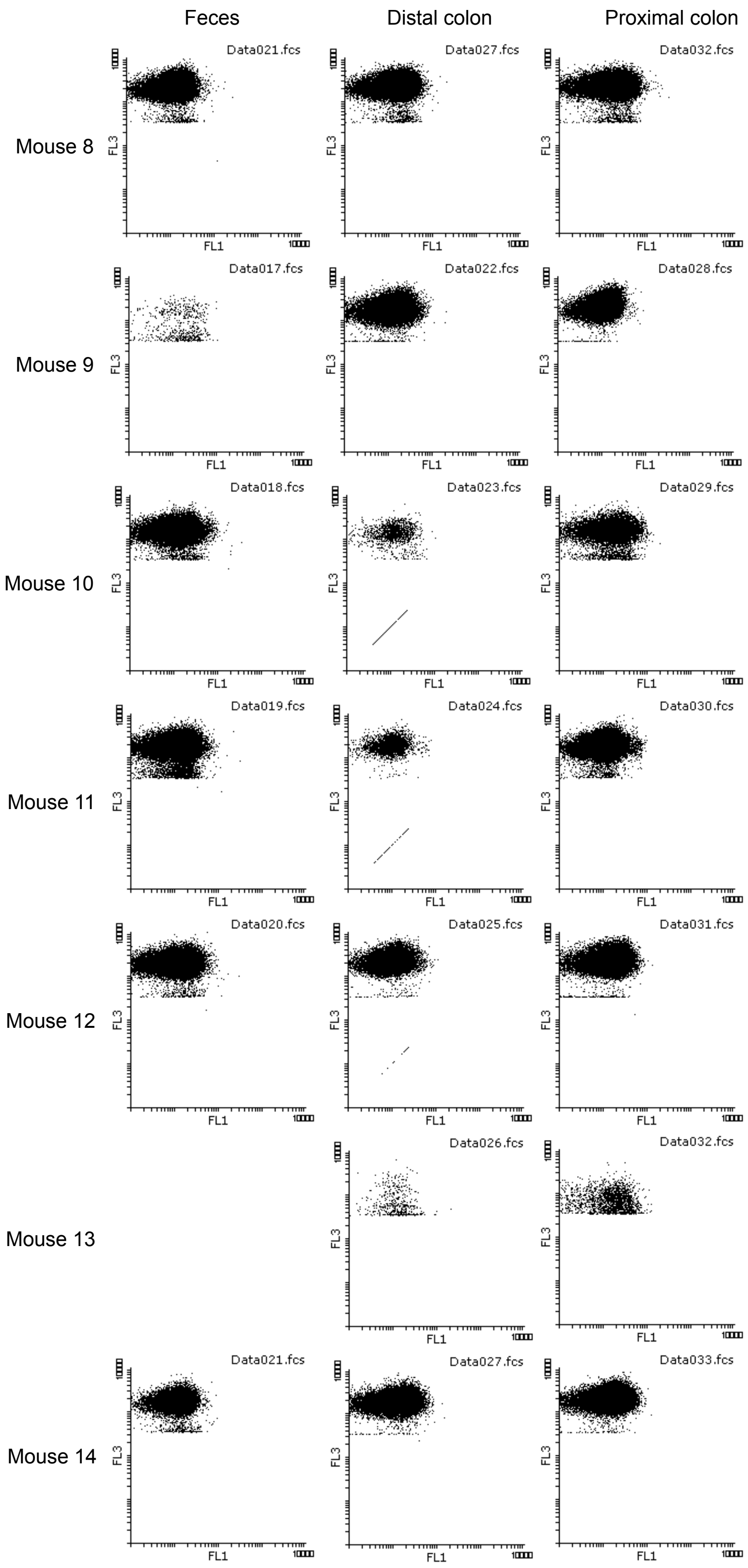
Feces
Distal colon
Proximal colon
Mouse 8
Data021.fcs
Data027.fcs
Data032.fcs
Mouse 9
Data017.fcs
Data022.fcs
Data028.fcs
Mouse 10
Data018.fcs
Data023.fcs
Data029.fcs
Mouse 11
Data019.fcs
Data024.fcs
Data030.fcs
Mouse 12
Data020.fcs
Data025.fcs
Data031.fcs
Mouse 13
Data026.fcs
Data032.fcs
Mouse 14
Data021.fcs
Data027.fcs
Data033.fcs
FL1
FL3

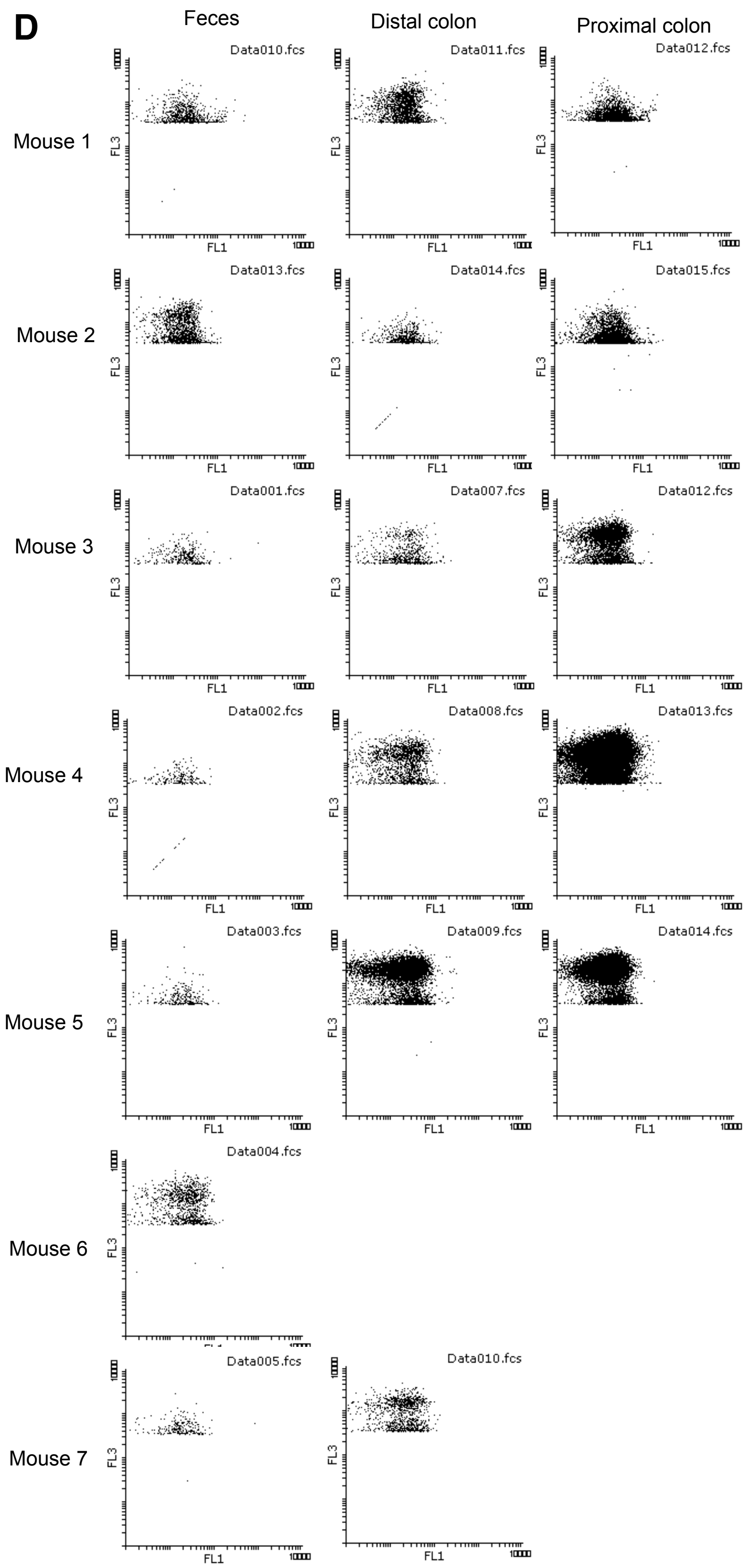

D
Feces
Distal colon
Proximal colon
Mouse 1
Mouse 2
Mouse 3
Mouse 4
Mouse 5
Mouse 6
Mouse 7
Data010.fcs
Data011.fcs
Data012.fcs
Data013.fcs
Data014.fcs
Data015.fcs
Data001.fcs
Data007.fcs
Data012.fcs
Data002.fcs
Data008.fcs
Data013.fcs
Data003.fcs
Data009.fcs
Data014.fcs
Data004.fcs
Data005.fcs
Data010.fcs
FL1
FL3

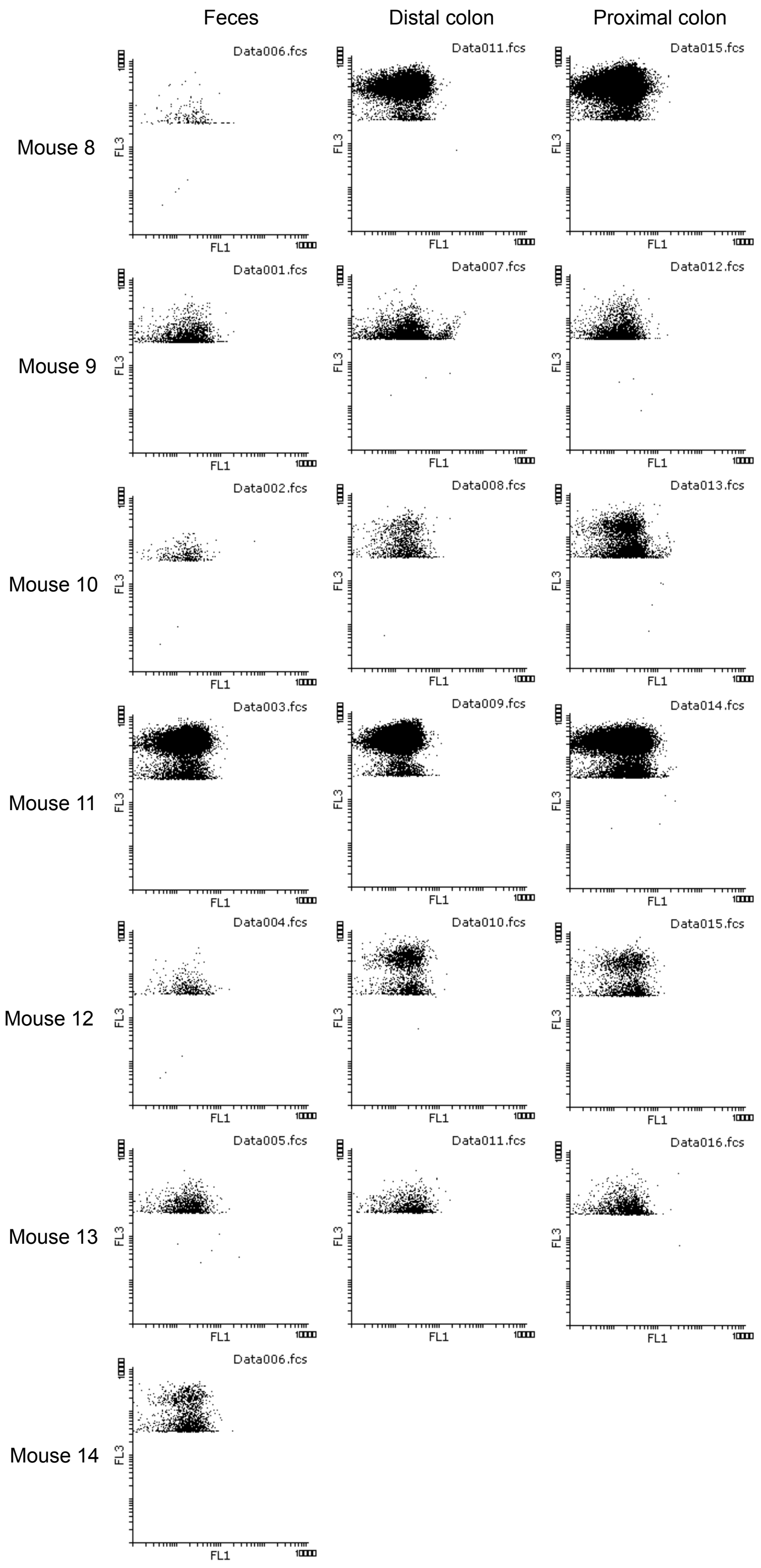
Feces
Distal colon
Proximal colon
Mouse 8
Data006.fcs
Data011.fcs
Data015.fcs
Mouse 9
Data001.fcs
Data007.fcs
Data012.fcs
Mouse 10
Data002.fcs
Data008.fcs
Data013.fcs
Mouse 11
Data003.fcs
Data009.fcs
Data014.fcs
Mouse 12
Data004.fcs
Data010.fcs
Data015.fcs
Mouse 13
Data005.fcs
Data011.fcs
Data016.fcs
Mouse 14
Data006.fcs
FL1
FL3

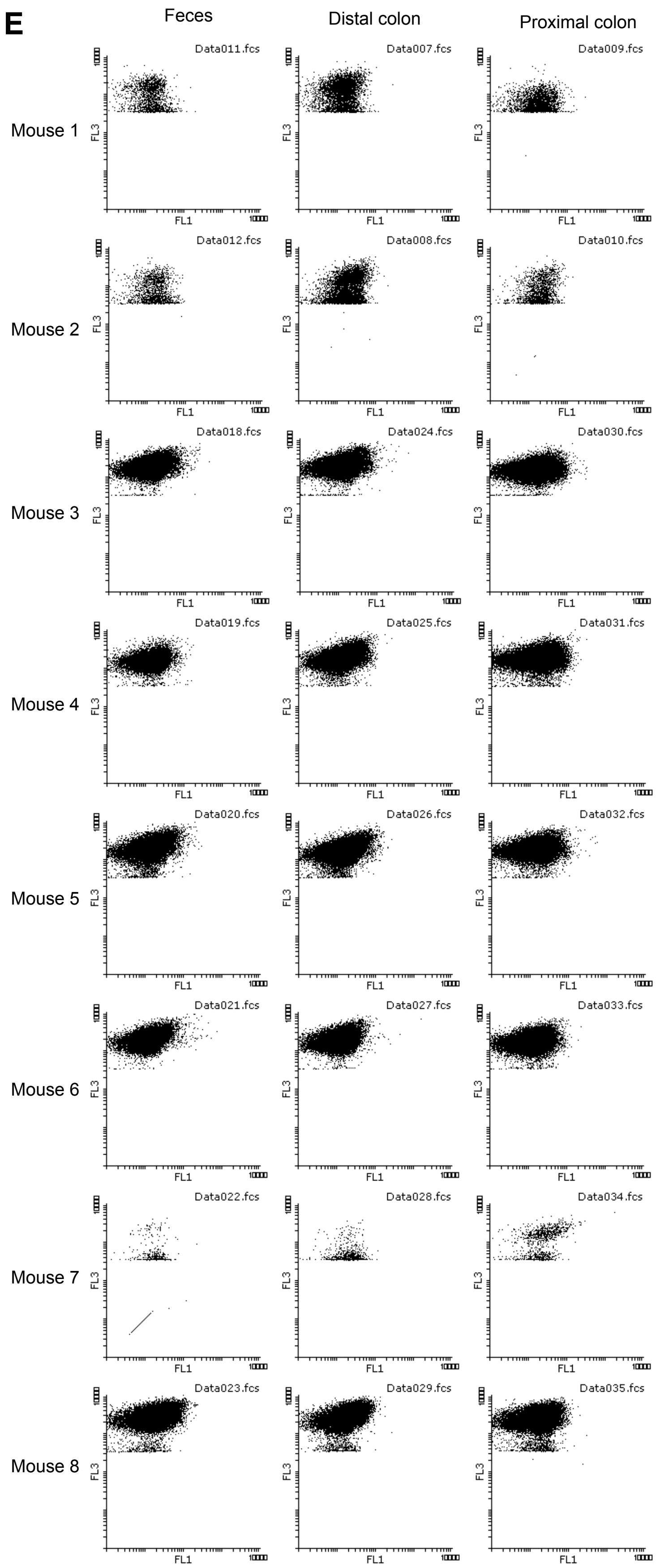
E
Feces
Distal colon
Proximal colon
Mouse 1
Mouse 2
Mouse 3
Mouse 4
Mouse 5
Mouse 6
Mouse 7
Mouse 8
Data011.fcs
Data007.fcs
Data009.fcs
Data012.fcs
Data008.fcs
Data010.fcs
Data018.fcs
Data024.fcs
Data030.fcs
Data019.fcs
Data025.fcs
Data031.fcs
Data020.fcs
Data026.fcs
Data032.fcs
Data021.fcs
Data027.fcs
Data033.fcs
Data022.fcs
Data028.fcs
Data034.fcs
Data023.fcs
Data029.fcs
Data035.fcs
FL1
FL3

F

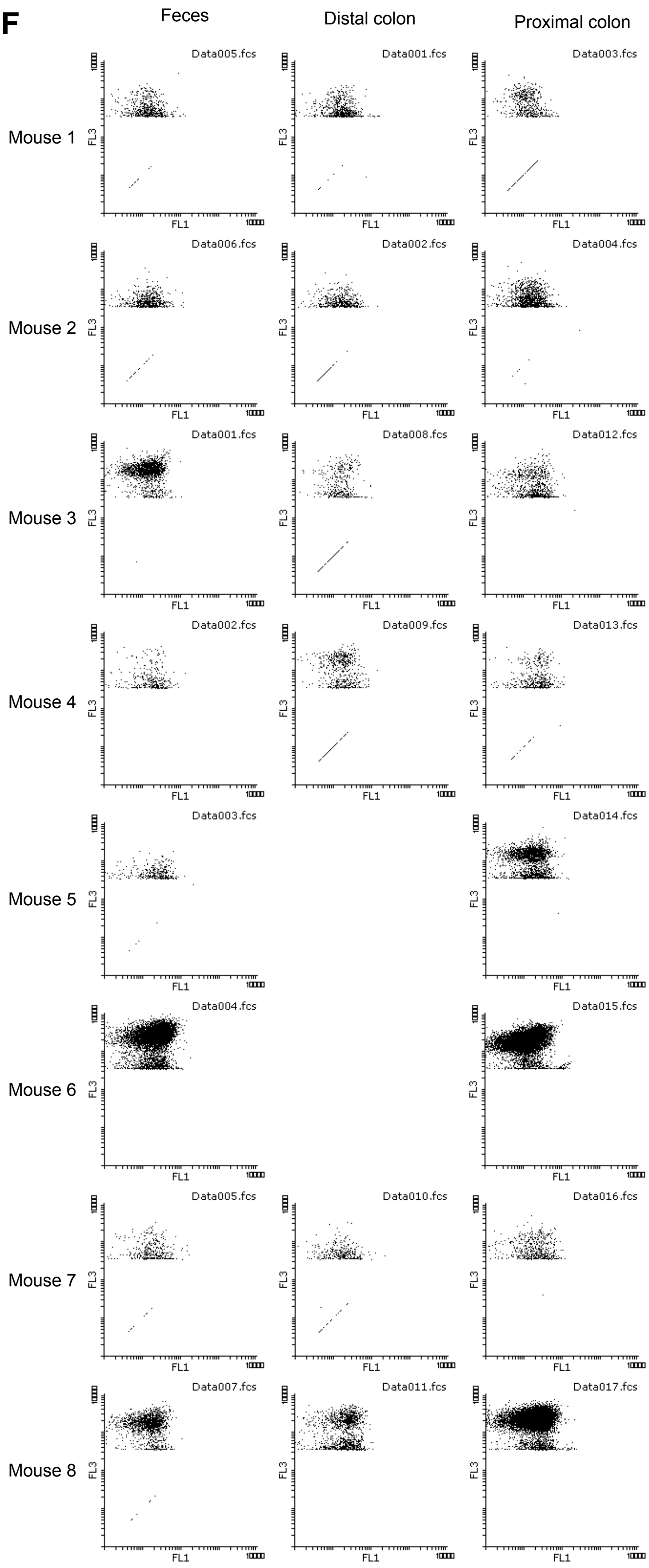
Feces
Distal colon
Proximal colon
Mouse 1
Data005.fcs
Data001.fcs
Data003.fcs
Mouse 2
Data006.fcs
Data002.fcs
Data004.fcs
Mouse 3
Data001.fcs
Data008.fcs
Data012.fcs
Mouse 4
Data002.fcs
Data009.fcs
Data013.fcs
Mouse 5
Data003.fcs
Data014.fcs
Mouse 6
Data004.fcs
Data015.fcs
Mouse 7
Data005.fcs
Data010.fcs
Data016.fcs
Mouse 8
Data007.fcs
Data011.fcs
Data017.fcs
FL1
FL3

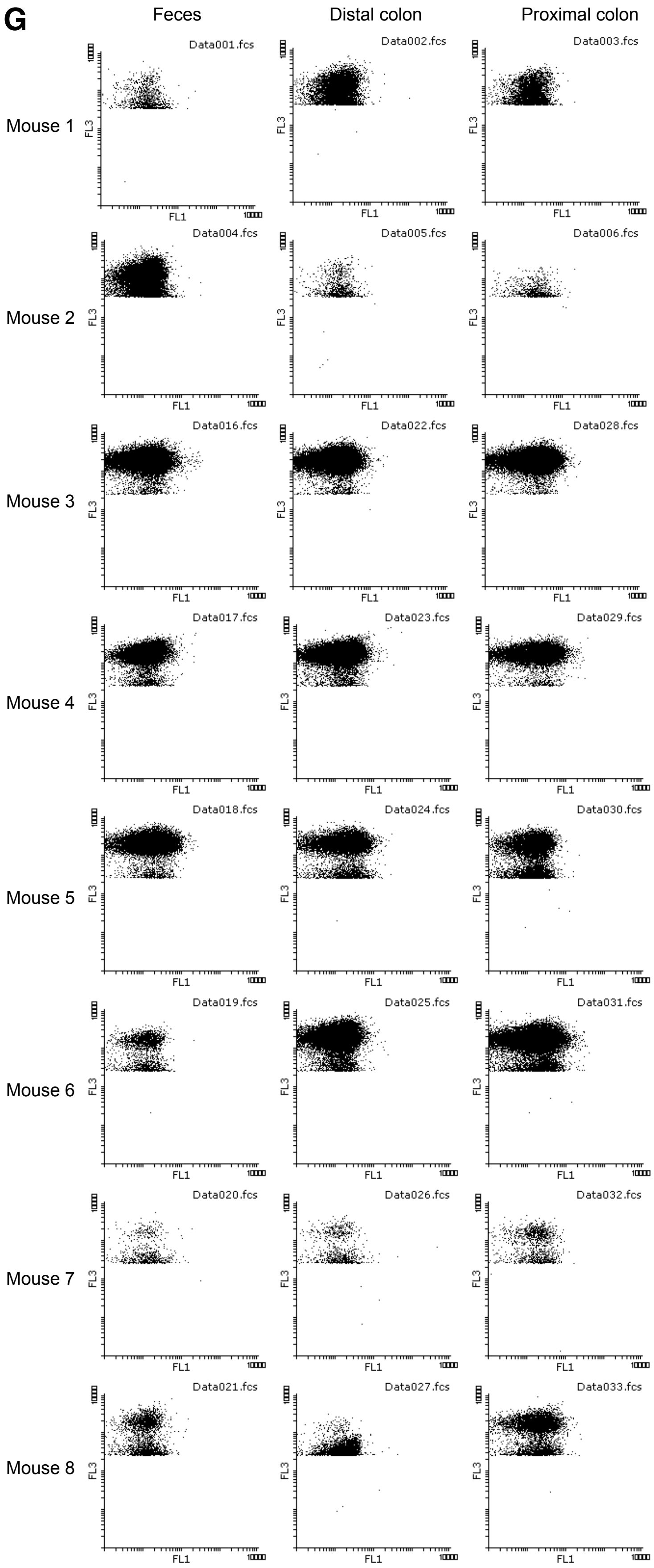

G
Feces
Distal colon
Proximal colon
Mouse 1
Mouse 2
Mouse 3
Mouse 4
Mouse 5
Mouse 6
Mouse 7
Mouse 8
FL1
FL3
Data001.fcs
Data002.fcs
Data003.fcs
Data004.fcs
Data005.fcs
Data006.fcs
Data016.fcs
Data022.fcs
Data028.fcs
Data017.fcs
Data023.fcs
Data029.fcs
Data018.fcs
Data024.fcs
Data030.fcs
Data019.fcs
Data025.fcs
Data031.fcs
Data020.fcs
Data026.fcs
Data032.fcs
Data021.fcs
Data027.fcs
Data033.fcs

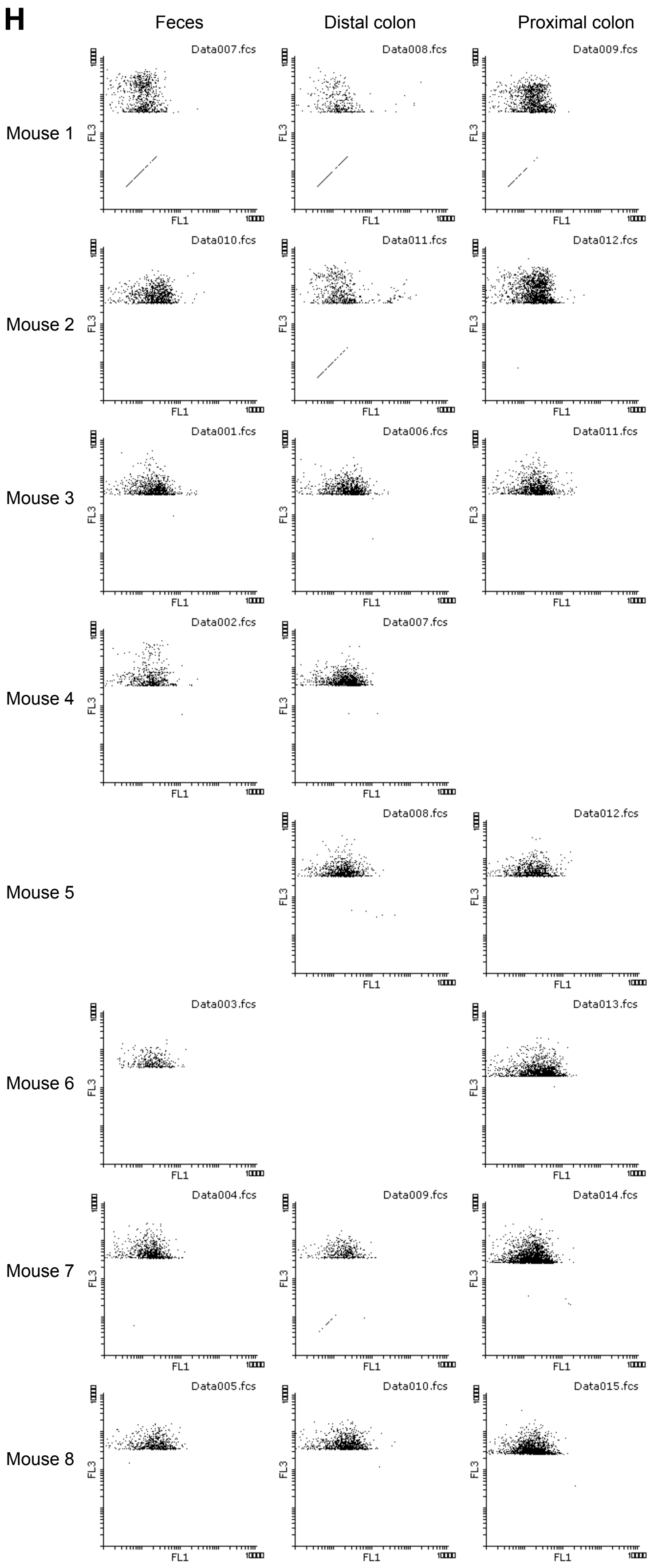
H
Feces
Distal colon
Proximal colon
Mouse 1
Mouse 2
Mouse 3
Mouse 4
Mouse 5
Mouse 6
Mouse 7
Mouse 8
Data007.fcs
Data008.fcs
Data009.fcs
Data010.fcs
Data011.fcs
Data012.fcs
Data001.fcs
Data006.fcs
Data011.fcs
Data002.fcs
Data007.fcs
Data008.fcs
Data012.fcs
Data003.fcs
Data013.fcs
Data004.fcs
Data009.fcs
Data014.fcs
Data005.fcs
Data010.fcs
Data015.fcs
FL1
FL3

Appendix Figure S25. 2D flow cytometry data. mCherry (FL3, y-axis) vs. sfGFP (FL1, x-axis) scatter plots for **(A)** ThsSR DSS-treated mice, **(B)** ThsSR untreated mice**, (C)** ThsSR D57A DSS-treated mice, **(D)** ThsSR D57A untreated mice, **(E)** TtrSR DSS-treated mice, **(F)** TtrSR untreated mice**, (G)** TtrSR D57A DSS-treated mice, **(H)** TtrSR D57A untreated m

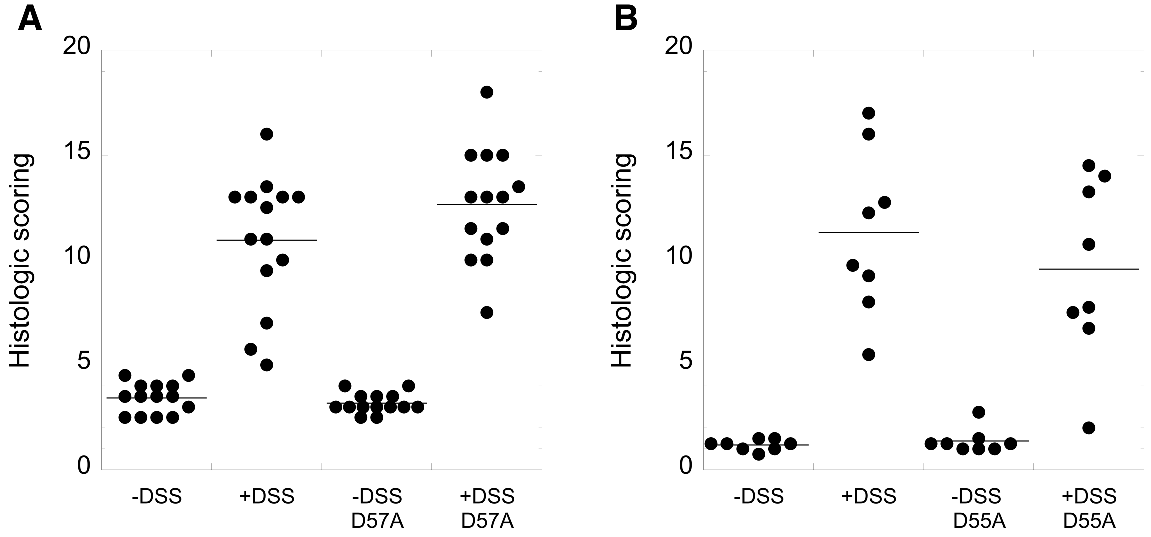

Appendix Figure S26. Histologic scoring of all mice in the healthy (-DSS) and DSS-treated cohorts for wild type and inactivated mutant **(A)** ThsSR and **(B)** TtrSR strains. Histologic scores are the average of two blinded histopathologists.

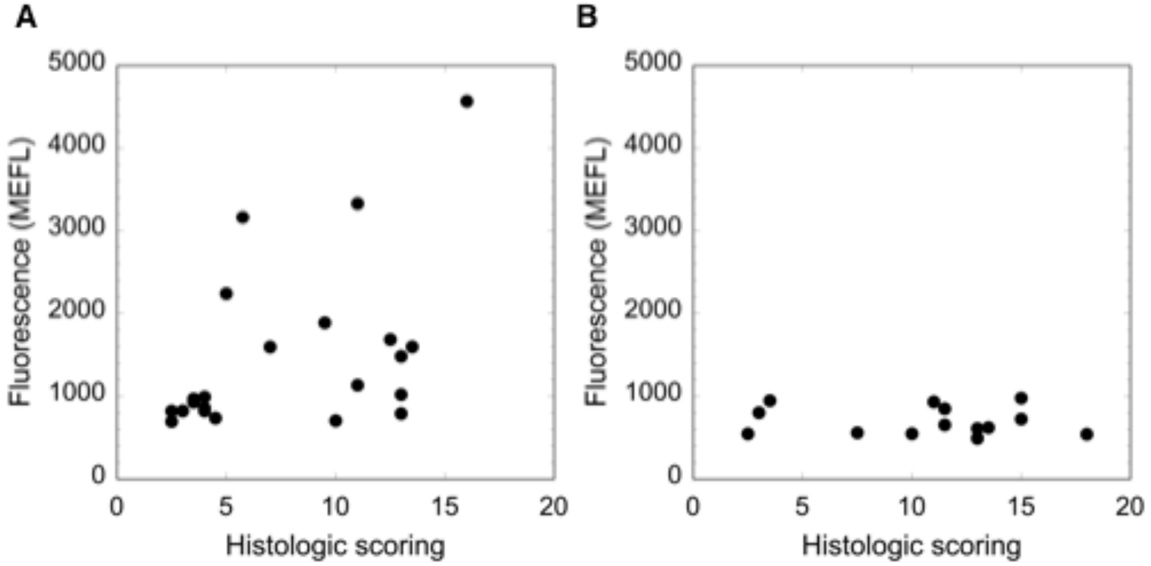

Appendix figure S27. Relationship between histologic score and fluorescence output of the **(A)** ThsSR and **(B)** ThsSR D57A sensors.

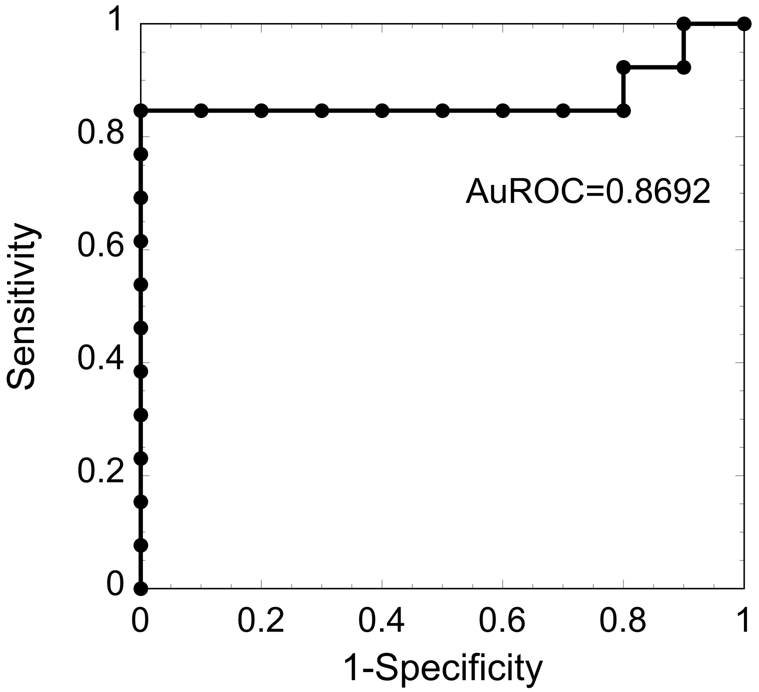

Appendix Fig S28. Receiver operating characteristic curve for ThsSR performance. The curve was generated as a diagnostic indicator of DSS treatment. The area under the curve (AuROC) is indicated.
